# Skull ecomorphological variation of narwhals (*Monodon monoceros*, Linnaeus 1758) and belugas (*Delphinapterus leucas*, Pallas 1776) reveals phenotype of their hybrids

**DOI:** 10.1371/journal.pone.0273122

**Published:** 2022-08-12

**Authors:** Deborah Vicari, Eline D. Lorenzen, Mikkel Skovrind, Paul Szpak, Marie Louis, Morten T. Olsen, Richard P. Brown, Olivier Lambert, Giovanni Bianucci, Richard C. Sabin, Carlo Meloro

**Affiliations:** 1 Research Centre in Evolutionary Anthropology and Palaeoecology, School of Biological and Environmental Sciences, Liverpool John Moores University, Liverpool, United Kingdom; 2 Natural History Museum of Denmark, Copenhagen, Denmark; 3 Globe Institute, University of Copenhagen, Copenhagen, Denmark; 4 D.O. Terre et Histoire de la Vie, Institut Royal des Sciences Naturelles de Belgique, Brussels, Belgium; 5 Dipartimento di Scienze della Terra, Università di Pisa, Pisa, Italy; 6 Department of Life Sciences, The Natural History Museum, London, United Kingdom; University College London, UNITED KINGDOM

## Abstract

Narwhals and belugas are toothed whales belonging to the Monodontidae. Belugas have a circumpolar Arctic and sub-Artic distribution while narwhals are restricted to the Atlantic Arctic. Their geographical ranges overlap during winter migrations in the Baffin Bay area (Canada/West Greenland) and successful interbreeding may occur. Here, we employed geometric morphometrics on museum specimens to explore the cranium and mandible morphology of a known hybrid (NHMD MCE 1356) and the cranium morphology of a putative hybrid (NHMD 1963.44.1.4) relative to skull morphological variation in the parental species. Specifically, we used 3D models of skulls from 69 belugas, 86 narwhals, and the two known/putative hybrids and 2D left hemi-mandibles from 20 belugas, 64 narwhals and the known hybrid. Skull shape analyses allowed clear discrimination between species. Narwhals are characterised by a relatively short rostrum and wide neurocranium while belugas show a more elongated and narrower cranium. Sexual size dimorphism was detected in narwhals, with males larger than females, but no sexual shape dimorphism was detected in either species (excluding presence/absence of tusks in narwhals). Morphological skull variation was also dependent on different allometric slopes between species and sexes in narwhals. Our analyses showed that the cranium of the known hybrid was phenotypically close to belugas but its 2D hemi-mandible had a narwhal shape and size morphology. Both cranium and mandible were strongly correlated, with the pattern of covariation being similar to belugas. The putative hybrid was a pure male narwhal with extruded teeth. Comparison of genomic DNA supported this result, and stable carbon and nitrogen isotope values suggested that the putative hybrid had a more benthic foraging strategy compared to narwhals. This work demonstrates that although the known hybrid could be discriminated from narwhals and belugas, detection of its affinities with these parental species was dependent on the part of the skull analysed.

## Introduction

The Monodontidae is a family of Odontoceti (toothed whales) that includes only two extant species: narwhals (*Monodon monoceros*, Linnaeus 1758) and belugas (*Delphinapterus leucas*, Pallas 1776) which have been found in northern Arctic waters since the early Pleistocene (2.58 Ma) [1]. Belugas are circumpolar species while narwhals are only found in the Atlantic region of the Artic.

The two species have an overlapping distribution in the Arctic during the winter migrations towards Disko Bay in Greenland [2, 3]. Repeated aerial surveys have shown that the beluga distribution has changed in relation to sea-ice cover [4]. This species generally starts southward migration during the summer (see **S1 Fig** for localities), departing from Canadian coasts, going to the west of Greenland during the autumn, moving into the north of Greenland for October, and to the south towards Disko Bay by December [4]. At the same time, narwhals from the west cost of Greenland (Uummannaq) start their southward winter migration towards Disko Bay [4]. This is an important feeding area where both species feed on Greenland halibut *Renhardius hippoglossoides*. It is also characterised by a sparse amount of open water, with no complete sea-ice coverage, and for this reason represents an area of contact between narwhals and beluga [4].

### Known hybrid

On 30^th^ March 1990 a narwhal/beluga hybrid (known as a “narluga”) skull was found at Disko Bay by researchers from Greenland Institute of Natural Resources [2] (**S1 File**), which showed features of both monodontid species, i.e., a wider and longer rostrum, and a number of horizontal teeth with a dental formula of 5-5/4-4 differing from narwhal and beluga (the first being virtually toothless except for the maxillary tusk present in males and sometimes found also in females while the second generally having a dental formula of 9-9/8-8). According to the Inuit hunter who claimed to know its origin, the hybrid whale was caught in mid-May 1986 or 1987, and it appeared to be a combination of the two species with a narwhal tail, and beluga pectoral fins with grey colouration [2]. The hybrid ancestry of this specimen has recently been confirmed by genomic analyses [5]. Narwhals and belugas are phylogenetically close [6], and shared a common ancestor in the late Miocene [1]. Natural hybridization is possible given that their distributions overlap temporally and spatially during the winter migration period [4, 7].

### Hybridisation in marine mammals

Cetaceans are karyologically uniform (2n = 44) with only seven species that deviate from this by showing a 2n = 42 [8–12] karyotype. This conservatism and the likelihood that they evolve relatively slowly at the molecular level, potentially allows greater hybridisation than observed in other mammals [13]. In addition, large seasonal migrations, synchronous breeding seasons [14], absence of marine geographical barriers and range changes due to global warming [15] may further promote hybridisation. There have been at least 57 described cases of hybridization in Cetacea (natural = 27, and captivity = 30) [16] across species and genera, involving 22 species, of which 14 are listed as endangered [7, 17]. Natural hybridisation has been described in mysticetes [17–19] and both natural and captive hybridisation has been described in odontocetes [13, 16, 17, 20–23].

With regard to other marine mammals, 1,144 natural and 7 captive hybridization events have been described within the Pinnipedia [16] among the Phocidae (true seals) [24, 25] and Otariidae (eared seals) [26–28], involving 13 species [16]. Unlike cetaceans, pinnipeds show a higher degree of variation in chromosome number (2n = 32 to 36) [29] which may explain why only three hybridization cases have been found between different genera [16].

### Hybridisation and morphology

Cetacean hybrids generally seem to show intermediate morphologies, with F1 (first generation) offspring displaying phenotypic characteristics of both parental species [21, 23, 30] even among different toothed whales classes. Most hybrids among marine mammals have been described morphologically [2, 13, 20, 31] although in recent years molecular techniques have been applied to identify the parental species [5, 13, 16, 26, 27, 32]. Interestingly, all cases of non-cetacean mammals hybridisation are found within genera [17, 32], which makes the inter-generic hybridization within cetaceans and pinnipeds quite unique.

Hybridisation can also affect diet and have long-term ecological and evolutionary consequences, depending on the mating system, hybrid frequency, and speciation process [32]. While differences in monodontid foraging ecology have been reported between sexes in West Greenland belugas and East Greenland narwhals [33], the ecomorphology of the only known hybrid is still poorly studied.

### Research goals

Geometrics morphometrics is an effective tool to identify hybrid morphologies and their parental species across a range of vertebrates [34–38]. Here, geometric morphometrics was applied to a sample of narwhal and beluga skulls to i) address the morphological identity of the known hybrid identified by [5] and an additional putative hybrid identified by DV, and ii) to investigate sexual shape (SShD) and sexual size (SSD) dimorphism in narwhal and beluga. In addition, we used genomic and stable isotope (*δ*^13^C and *δ*^15^N) analyses of the putative hybrid to support our morphological findings and to gain insights into its foraging strategy.

## Material and methods

### Sample size

**Fig 1:**
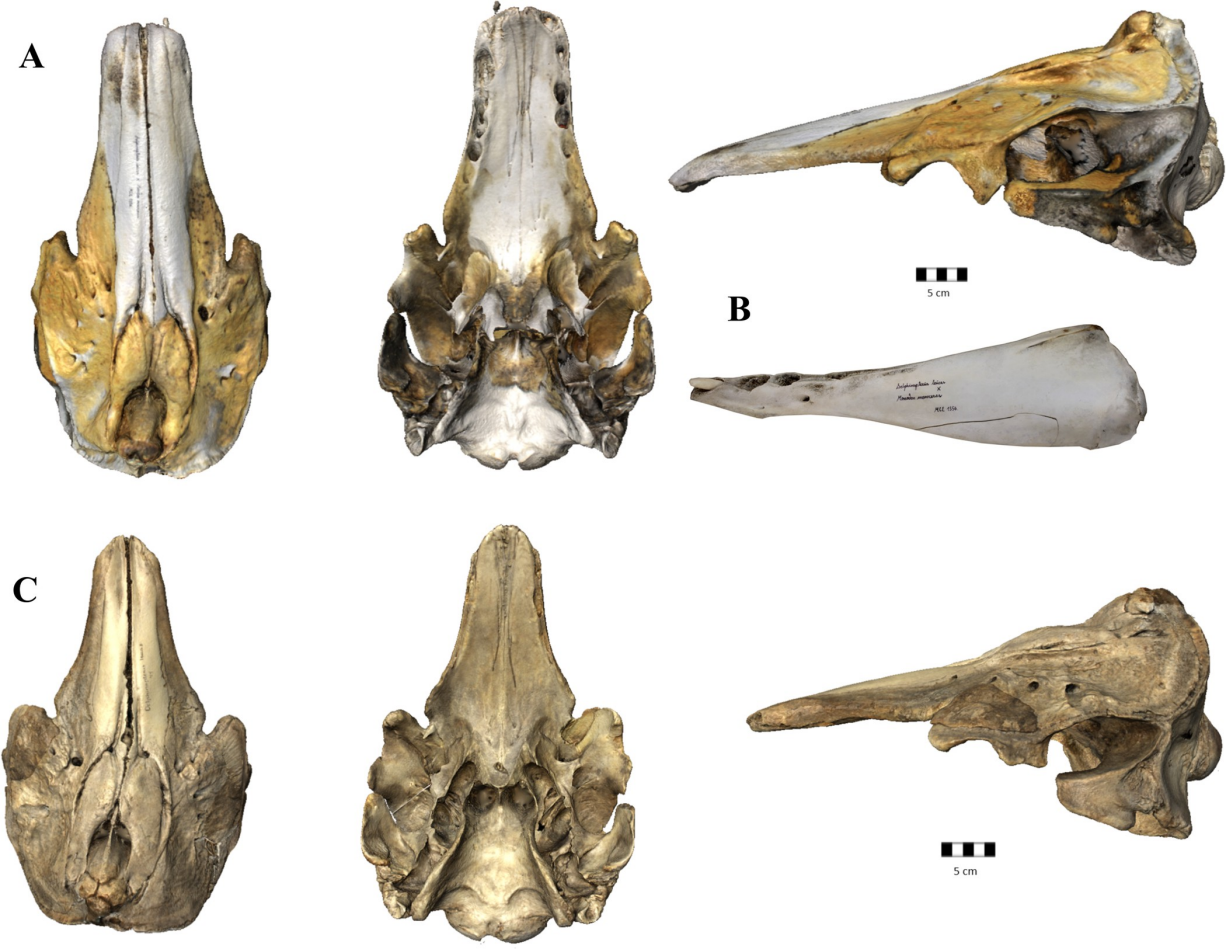
Known hybrid and putative hybrid specimens. 3D models of the crania of (A) the known hybrid MCE 1356 (narluga) in dorsal, ventral, and left lateral view. (B) Photo of the left hemi-mandible of the narluga in lateral view. (C) Cranium of the putative hybrid specimen NHMD 1963.44.1.4 in dorsal, ventral, and left lateral view. Scale bar 5cm.

We analysed 157 monodontid crania including 86 narwhals, 69 belugas, the known hybrid and one putative hybrid (i.e., specimen NHMD 44.1.4.1963, which was detected by the first author of this study during data collection) and 85 left hemi-mandibles including 64 narwhals, 20 belugas, and the known hybrid hemi-left mandible (**Fig 1**) from the collections at: Natural History Museum of Denmark (NHMD), Natural Museum of Scotland (NMS), La Specola, Florence (Italy) (**S1 Appendix** -List of specimens). The putative hybrid was labelled *Delphinapterus leucas* in the collection, but both narwhal-like and beluga-like characteristics were noted (i.e., narwhal cranial shape and two upper row erupted teeth like belugas). All crania analysed were adult specimens, as the maxillary bones reached caudally the nuchal crest and part of the frontal bone was not visible [39].

### Cranium landmarking

**Fig 2:**
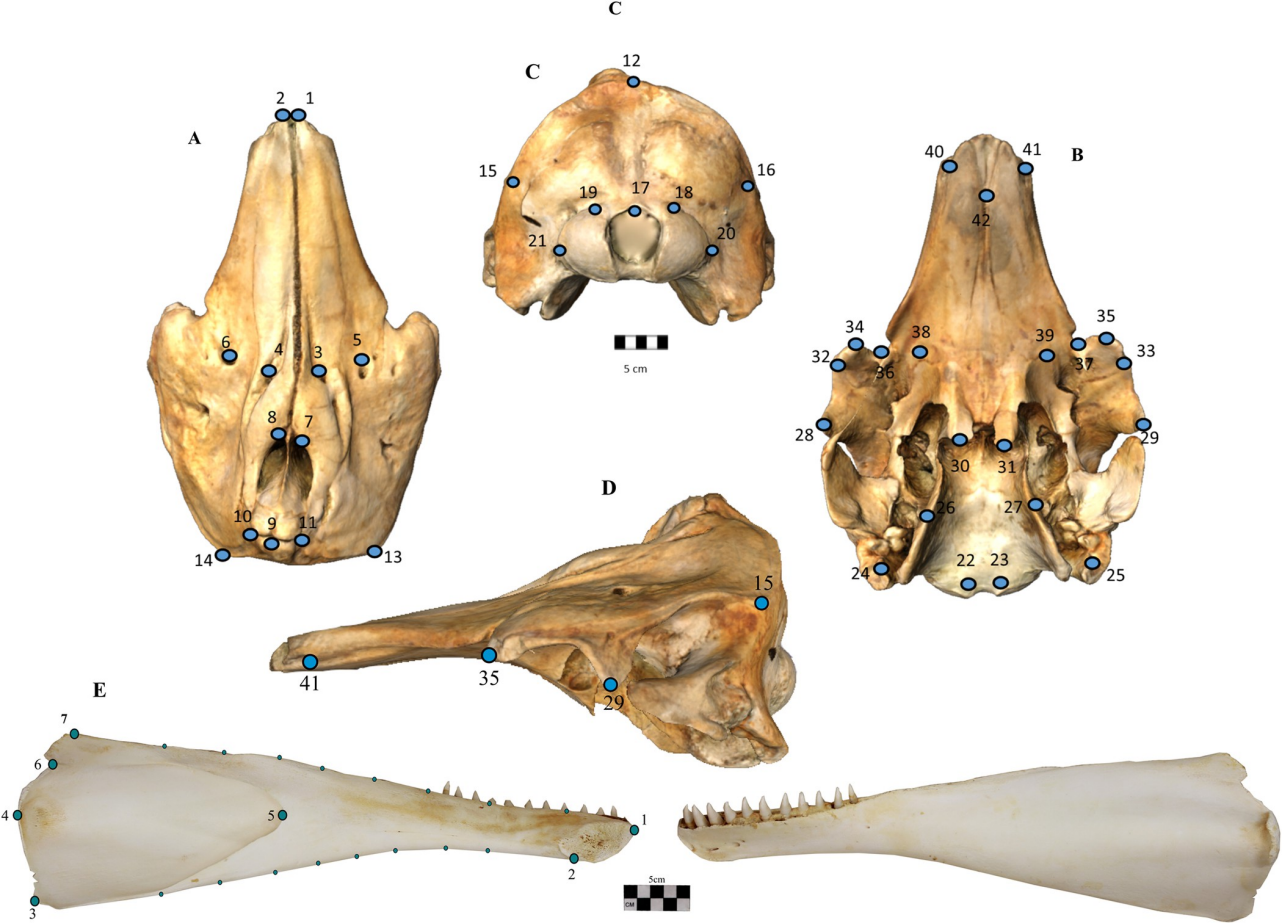
Landmark location in cranium and mandible. Landmark configuration on photogrammetric-based 3D model specimen for the cranium of a narwhal (*Monodon monoceros* NHM 1937.10.30.2), in (A) dorsal, (B) ventral, (C) occipital and (D) left lateral view. (E) The position of the 7 anatomical landmarks, and 16 semilandmarks on a beluga left hemi-mandible (*Delphinapterus leucas* NHMN 1098) in lingual view. The labial view of the hemi-mandible is also provided. Scale bar 5 cm.

We collected 42 three-dimensional landmarks which are representative of both dorsal and ventral sides of the cranium (**Fig 2**; **Table 1**) using a Microscribe digitizer. Most of these landmarks were type 2 (i.e., landmarks on the maximum or minimum curvature of a structure [40]) and showed good repeatability. Due to the large size of the specimens, two landmarking sessions for each specimen were recorded to cover both dorsal and ventral parts. These were then merged using the DVLR (Dorsal-Ventral-Left-Right fitting, www.nycep.org/nmg) software. Due to missing landmarks in 40 narwhal and 30 beluga specimens (note that the putative hybrid had missing landmarks on the pterygoids while the known hybrid cranium was complete) the function *estimate*.*missing* in the R package geomorph 3.1.2 package [41, 42] was used within each species group. Missing landmarks on the putative hybrid specimen (labelled as *D*. *leucas* in the collection) were estimated within the narwhal group, as DNA analysis of this specimen suggested it was a pure narwhal (see below).

**Table 1:** Description of the 42 homologous landmarks used in the geometric morphometric analysis of 157 monodontid skulls.

No.	Homologous landmarks on the cranium
**1–2**	Tip of the rostrum
**3–4**	Anteriormost point of the premaxillary foramen
**5–6**	Anterior dorsal infraorbital foramen
**7–8**	Anteromedial point of the external bony nares
**9**	Anteriormost point of the medial suture between the nasal bones
**10–11**	Sutural triple-junction between nasal, frontal and maxilla
**12**	External occipital protuberance
**13–14**	Sutural triple-junction between supraoccipital, frontal and parietal
**15–16**	Posteriormost point on the temporal crest
**17**	*Opisthion*; middle point of the dorsal border of the *foramen magnum* on the intercondyloid notch
**18–19**	Dorsal tip of the occipital condyle
**20–21**	Lateral tip of the occipital condyle
**22–23**	Ventral tip of the occipital condyle
**24–25**	Medial tip of the paroccipital process of the exoccipital; ventralmost point of the paroccipital process
**26–27**	Suture of pterygoid and basioccipital at the junction between pharyngeal crest and basioccipital crest
**28–29**	Posteroventral point of the postorbital process of the frontal
**30–31**	Pterygoid *hamulus*; posterior margin of the hard palate and the border of the internal bony nares
**32–33**	Anteroventral point of the preorbital process of the frontal
**34–35**	Anterior tip of lacrimal bone
**36–37**	Antorbital notch
**38–39**	Anteriormost point of the palatine
**40–41**	Posteriormost point of the upper alveolar groove
**42**	Medial junction of vomer and premaxilla

The landmark scheme used in this study was assessed using the Landmark Sampling Error Curve, (LaSEC) function within the LaMBDA package [43]. This function subsamples the dataset by randomly selecting three landmarks and generating a median fit obtained by Procrustes distance between the original dataset and the subsampled one per each selected number of iterations. This demonstrated that 19 landmarks provide a median fit value of 0.90, 26 provide a median fit value of 0.95, and that our 42 landmark scheme was dense enough to achieve a fit value of 1 which indicates that they suitably characterised both monodontids cranial shape and size.

### Mandible landmarking

Mandibles were photographed in lateral view at a standard distance using a Canon EOS 1100D digital camera (f/8, ISO 100, focal length = 37mm). The left hemi-mandibles represented a subset (n = 45) of the individuals on which crania were measured. To increase the sample size, we also included hemi-mandibles from specimens with no cranial data (n = 40).

We recorded 23 two-dimensional landmarks on the 85 hemi-mandibles using the software TPSDig [44]. Seven of these were homologous landmarks as described in [45] and 16 were semi-landmarks (**Fig 2E**; see **Table 2** for landmark description). As the anteriormost point of the internal mandibular foramen in the hybrid mandible was not complete, this landmark was excluded from the analyses. A scale bar next to the specimen ensured scaling of each digital image. Slider files were prepared with TPSUtil to indicate the semi-landmarks, and a Generalized Procrustes Analysis (GPA), with sliding of semi-landmarks [40] was performed in TPSRelw. The aligned specimens were subsequently imported into R [46].

**Table 2:** Description of the 7 homologous landmarks used in the geometric morphometrics analysis of 85 monodontid 2D hemi-mandibles.

No.	Homologous landmarks on the lingual view of the hemi-mandible
**1**	*Pogonion*; Tip of the mandible
**2**	*Gnathion*, the lowest point of the midline of the mandibular symphysis
**3**	Posterior ventral tip of the angular process
**4**	Posteriormost point of the condyle
**5**	Anteriormost point of the internal mandibular foramen
**6**	Most concave point of the mandibular notch
**7**	Dorsal tip of the coronoid process
**8–15**	Semilandmarks curve describing the mandible body and coronoid crest curvature
**16–23**	Semilandmarks curve describing the base of the mandible

### Geometric morphometrics

Generalised Procrustes Analysis (GPA) was used to analyse raw landmark coordinates from crania and mandibles, separately, [47] using the geomorph 3.1.2 package [41, 42, 48]. As the known hybrid and the putative hybrid differ from their parental species in having a dental formula of 5-5/4-4 and 3–3 (only cranium) respectively, we ran the analyses on two datasets: Dataset 1- with 42 landmarks and Dataset 2 with 40 landmarks (without landmarks 40 and 41, dealing with the posterior extent of the alveolar groove).

Shape coordinates were subsequently analysed with Principal Component Analysis (PCA). Variation in skull shape along the Principal Component (PC) axes was visualised using warping of 3D models already available to us via photogrammetry, while mandible shape changes from the mean were described using Thin Plate Spline (TPS) [40].

Procrustes ANOVA (function “procD.lm” in the geomorph package described previously) was used to i) test for morphological changes related to allometric variation [49] in the cranium and mandible complete datasets including and excluding the hybrid and putative hybrid, ii) evaluate SSD and SShD in a subset of sexed cranial specimens (24 belugas: ♀ = 11, ♂ = 13; 49 narwhals: ♀ = 25, ♂ = 24) excluding the hybrid and putative hybrid, and iii) explore allometric slopes within each sexed species in the sexed cranial dataset to reveal differences in cranium growth rate and morphological changes with cranial size between and within species.

### Classification tests for cranium and mandible

To identify phenotypic similarities between the hybrid and putative hybrid specimens and their parental species we adopted multiple statistical approaches.

First, we applied Unweighted Pair Group Method with Arithmetic mean (UPGMA) clustering to Procrustes distances (separately on both the cranium and mandible datasets) to provide a visual representation of the clustering of specimens without any *a priori* grouping [50, 51] using PAST 2.17c [52].

Second, we employed Discriminant Function Analysis (DFA) in SPSS 25.0 [53] to identify vectors that maximise differentiation between known belugas and narwhals [49]. PC vector scores were input into the DFA. To reduce data dimensionality, we adopted a stepwise method that is effective with shape variables [54, 55]. Significant PC vectors were retained based on the *F*-values in the MANOVA and added into the DFA to enhance discrimination between groups. Percentage of correctly classified cases for each species was assessed after cross-validation and group membership of the known and putative hybrid specimens was assigned based on the significant DF scores.

Third, we adopted a *k*-means clustering algorithm with *k* = 2 in order to assign, based on shape variables only, individuals to one species or another (program: PAST 2.17c [52]). In this analysis, individuals are divided into *k* groups so that members of one group are more similar to each other, minimizing within group variation [56]. The analysis proceeds in an iterative manner: first, the centroids of *k* groups are calculated and each specimen is then associated with the closest centroid. Second, a new *k* centroid is calculated. The procedure ends when the assignments of individuals to groups does not change with further iterations. This analysis differs from the DFA as groups are not specified *a priori*, and the clusters are formed considering similar shape variables only.

### Skull integration

Patterns of covariation between cranium and mandibular shape were explored using a two-blocks Partial Least Squares (2B-PLS) analysis [57] on a dataset of 47 complete skulls (12 belugas, 34 narwhals and 1 known hybrid). Covariation between the cranium and mandible would confirm if each species is driven by similar allometric pattern and ecological specialization (same niches). 2D mandible coordinates were transformed into 3D coordinates by adding the z axis with a score of 0.0. PLS is useful for studies of integration between two blocks of variables [49, 58–60]. Differences in covariation trajectories between species were tested using comparison of the angles of the PLS vectors using MorphoJ 1.06d [60, 61] against the null hypothesis of no difference from two random orthogonal vectors (90°). Therefore, a significant *P* value will reflect a statistically more similar shape variation than two random vectors. In contrast, a non-significant *P* value will indicate different directionalities in shape between species. PLS analyses were applied to the narwhal and the beluga datasets separately, and the angle between each PLS of each dataset was calculated. This provides an indication of any dissimilarities in morphological integration between the two species. Like the PCs in the PCA, the singular axes (SAs) in the PLS can be described by deformations along axes, helping with the interpretation of the results [49]. Statistical significance was tested with a permutation test that examined the null hypothesis of no covariation between cranium and mandible [49].

### Genomic analysis of the putative hybrid specimen

To identify the genetic ancestry and sex of the putative hybrid, we generated genome-wide genetic data and compared it to a reference panel of eight narwhals and eight belugas. This was done by extracting bone powder from the specimens and following the methodology described by [5]. The known hybrid had already been analysed with this technique and was shown to be a male specimen of first generation offspring hybrid [5]. The DNA extraction procedure and data processing are detailed in full in **S2 File**.

The putative hybrid had a genome wide coverage of 0.93x. The belugas and narwhals in the reference panel had an average nuclear genome-wide coverage of 0.25x and 0.19x, respectively. Analyses were performed on two datasets: i) the complete dataset which included all sites that passed basic quality filters (4,281,320 sites), and ii) the fixed-sites dataset which was further filtered to only include sites fixed for alternate alleles in narwhals and belugas reference panel (75,362 sites). We included admixture analyses using NGSadmix [62] and fastNGSadmix [63] as well as PCAs using PCAngsd [64]. We also constructed a haplotype network from an alignment of mitochondrial genomes of the putative hybrid and the narwhal and beluga reference panel, following the methodology outlined in [5].

### Stable carbon and nitrogen isotope extraction

To investigate the feeding ecology of the putative hybrid, we generated bone collagen δ^13^C and δ^15^N data for the specimen following the protocol in [33]; δ^13^C indicates feeding habitats (pelagic vs benthic, offshore vs coastal), δ^13^N reflects the trophic level [33]. The data were analysed with published bone collagen δ^13^C and δ^15^N values already available for a subset of the cranial specimens analysed in our study (45 narwhals, 13 belugas; 1 known hybrid), published by [5] and [33]. The putative hybrid became part of the NHMD in 1963, but there is no information on when it was collected. Hence, we adjusted the carbon value of the specimen for the Seuss effect assuming a date of 1963 using the SuessR [65] package in R software; the Seuss effect explains the change in ratio of carbon isotope in the atmosphere due to the industrialization that has occurred since the late 19th century [66, 67], and the Suess corrected values were used for the narwhal/beluga stable isotope reference panel in the analysis. To test for dietary differences between narwhals and belugas we compared their δ^13^C and δ^13^N using unpaired t-tests.

## Results

### Cranium

#### Dataset 1: 42 landmarks

The PCA analysis of shape data is shown in **S2 Fig**; **S2 File**. PC1 and PC2 together accounted for 77.3% of the total variance. In **S2 Fig**, the known hybrid is closer to beluga morphospace while the putative hybrid occupies the middle part of the morphospace between the two species groups.

#### Dataset 2: 40 landmarks

**Fig 3:**
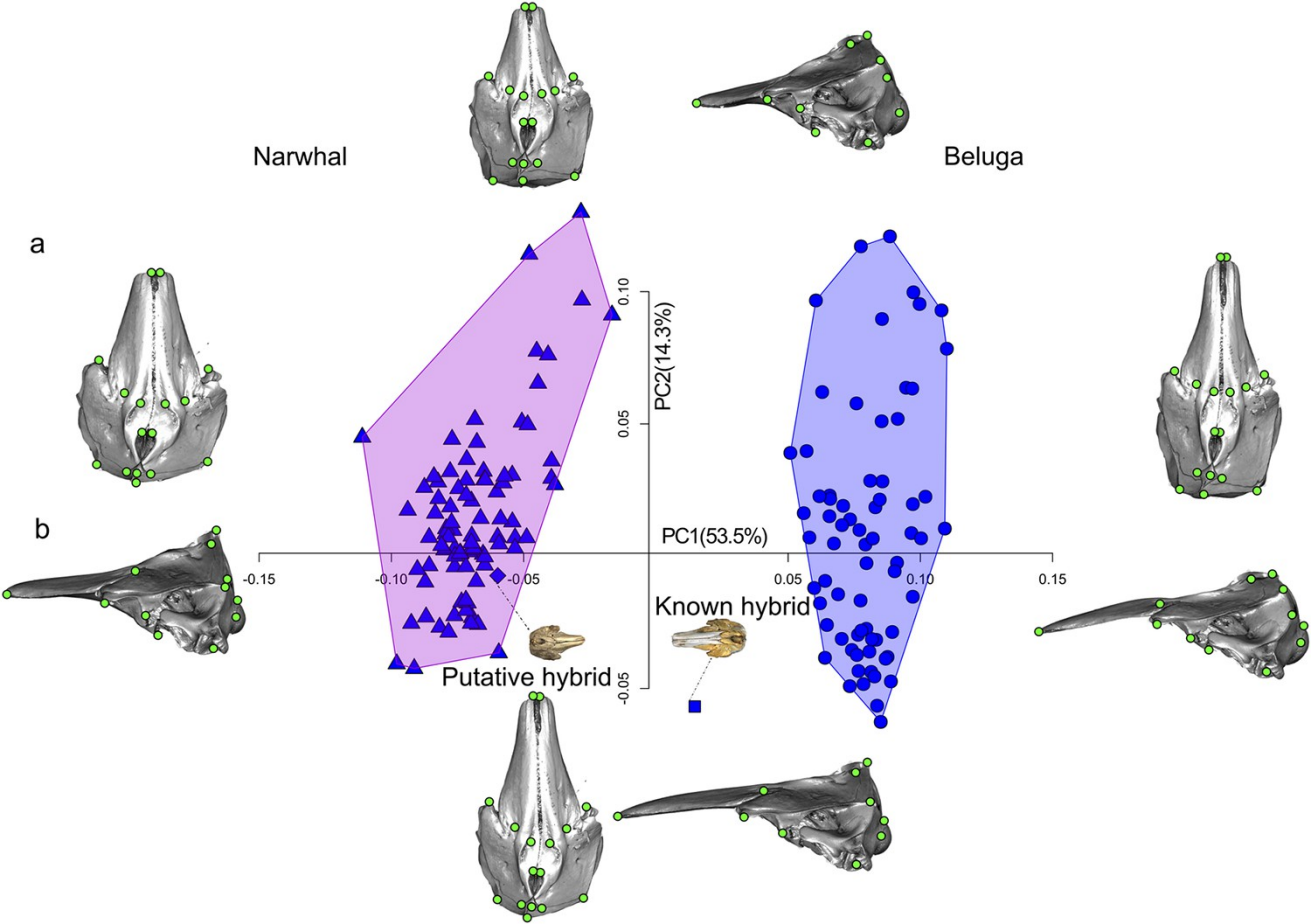
PCA on crania dataset with 40 landmarks. Principal Component (PC) plot of the cranial shape of the three-dimensional (3D) Monodontidae dataset with 40 landmarks. Shape differences along the axis of the PC1 and PC2 are visualized with warping in A) dorsal and B) left lateral view.

PC1 explains 53.5% of variation while PC2 explains 14.3% (**Fig 3**). Positive PC1 scores reflected lateral compression of the maxilla and premaxillary bones. Negative scores on this axis reflected a broader braincase and a shorter nuchal crest, resulting in a spindle-shaped cranium. The area of the occipital condyles described by seven landmarks (LM 17, 18, 19, 20, 21, 22, 23 in **Fig 2**), assumes a narrower shape compared to PC1 negative scores. The occipital condyles are located lower on the cranium. Landmarks on the pterygoid *hamulus*, which delimits the posterior margin of the hard palate, and the border of the internal bony nares diverge from the sagittal plane in the negative scores while they converge towards this plane in narwhals (negative scores). Also, the landmarks on the teeth shift forwards in the narwhals due to absence of maxillary teeth and to the presence of the maxillary tusk that only erupt on the left side of the cranium in males of this species.

PC2 describes the changes in the length of the rostrum, the outline of the temporal area and the concavity of the profile of the facial region. Negative values of PC2 reflect a shrinking and lengthening of the temporal fossa which is the site of origin of the temporal muscles, which are the main adductors of the mandible. These negative values show the dorsal displacement of the unpaired landmark (LM 12 in **Fig 2**) on the nuchal crest. Positive values of PC2 describe a shortening of the pterygoids and forward shift of the nasal area together with a forward shift of the landmarks describing the ventral most point of the paraoccipital process and the beginning of the alveolar groove.

These PCs were very similar to those obtained from the dataset with 42 landmarks, but with one main difference: the putative hybrid clearly clustered with narwhals. Because this configuration is less affected by landmarks taken on the posterior most point of the alveolar groove, it was used in all subsequent analyses.

#### Allometry and sexual dimorphism

**Fig 4:**
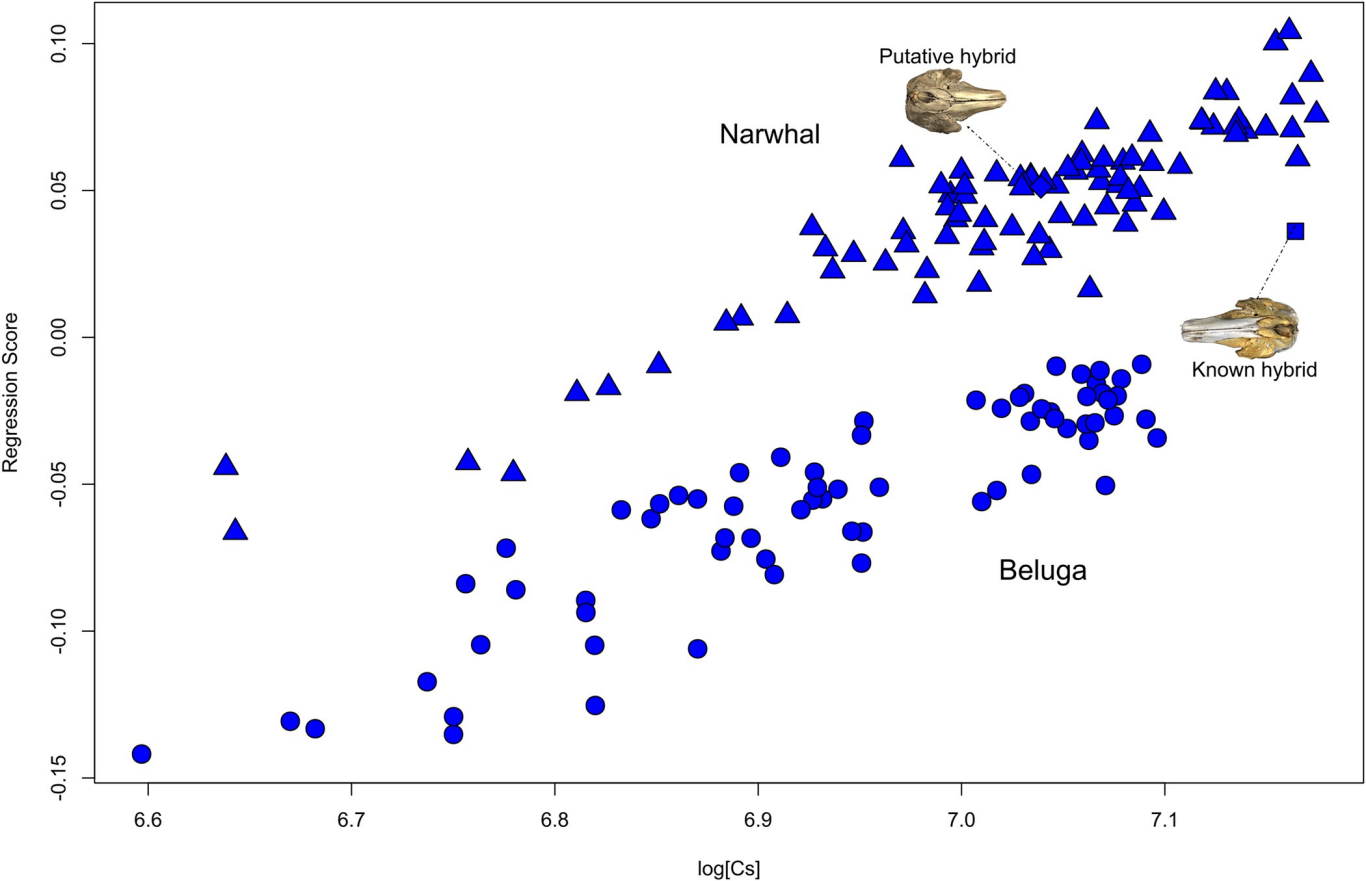
Allometry in cranial dataset with 40 landmarks. Regression of the cranial shape component against log CS for both narwhal (triangle) and beluga (circle) shows cranial shape changes in relation to size. The known hybrid is represented by a square, and the putative hybrid by a diamond shape. The dotted line shows the known hybrid and putative hybrid cranium.

Size significantly influenced cranial shape (P < 0.001) in the complete (*n* = 157) and reduced (i.e., without known and putative hybrid; *n* = 155) datasets with log CS explaining 26.6% and 27.1% (**Table 3A**) of shape variance, respectively. The known hybrid is much larger than the putative hybrid specimen, which falls clearly within the narwhal size range (**Fig 4; S3 File**). ‘Species’ makes a significant contribution to cranial shape variation (48.1%) and its interaction with size shows that allometric slopes differ between the narwhal and the beluga (**Table 3A**). When only sexed individuals were analysed (*n* = 73), the differences between species are more pronounced (with species explaining 67.2% of the variation in shape and 25.4% of the variation in size) although sex is only significant for cranial size (**Table 3B, 3C**).

**Table 3:** Procrustes ANOVA to test for A) allometry in 155 monodontids excluding the known and putative hybrid; B) sexual shape dimorphism (SShD) and C) size (SSD) dimorphism on crania shape of 73 sexed monodontid specimens.

		Df	SS	MS	Rsq	F	Z	P
**A) Monodontids Allometry**	**Cs**	1	0.6360	0.6360	0.2707	170.7967	4.1556	**0.001**
	**Species**	1	1.1288	1.1288	0.4805	303.1561	3.3558	**0.001**
	**Cs:Species**	1	0.0218	0.0218	0.0092	5.8586	6.3622	**0.001**
	**Residuals**	151	0.5622	0.0037	0.2393			
	**Total**	154	2.3489					
**B)**	**Sex**	1	0.0124	0.0124	0.0122	2.7289	1.6559	0.071
	**Species**	1	0.6818	0.6818	0.6723	149.3475	6.26	**0.001**
**Shape**	**Sex:Species**	1	0.0048	0.0048	0.0047	1.0541	0.3319	0.354
	**Residuals**	69	0.3150	0.0045	0.3106			
	**Total**	72	1.0141					
**C)**	**Sex**	1	0.1120	0.1120	0.0996	10.7377	2.5702	**0.001**
	**Species**	1	0.2858	0.2858	0.2543	27.3868	3.7556	**0.001**
**Size**	**Sex:Species**	1	0.0057	0.0057	0.0051	0.5512	0.1108	0.470
	**Residuals**	69	0.7201	0.0104	0.640			
	**Total**	72						

Splitting the sample by species shows allometry to have a similar impact on cranial shape for the narwhal (27.1% of variation) and the beluga (28.0% of variation). Sex is not significant for cranial shape for either species when analysed separately, although allometric slope differences occur between sexes for narwhals (**S4 File**). Sex is significant for cranial size for narwhals (F_2,49_ = 11.49, P = 0.004; **S4 File**) explaining 19.6% of variance, but not for belugas (F_2,24_ = 2.37, P = 0.153; **S4 File**) where it explains only 7.6% of the variance.

#### Classification test based on the cranium

UPGMA phenograms (**S3 Fig**) preserve small-scale distances very well and so here neatly summarize shape relationships. Members of each species cluster together, while the putative hybrid falls into the narwhal group, and the known hybrid falls within the beluga cluster at node 54, but outgroups the specimens descending from the two clusters at node 56 and 57. Cophenetic correlation for this UPGMA tree is quite high (*r* = 0.9652), showing that it provides a good reflection of pairwise distance structure.

After stepwise procedure, only 10 out of 113 shape PC vectors and log CS were selected by the DFA. One significant discriminant function was extracted to differentiate species (*Wilk’s lambda* = 0.155, *χ2* = 283.541, *df* = 2, P < 0.001). It correctly classified 98.1% of the specimens and assigned the known hybrid to the beluga group and the putative hybrid to the narwhal group. The beluga group was classified as 95.7% beluga and 4.3% narwhal, while the narwhal group was 100% in the narwhal group. This prediction is equally confirmed by the *k*-means clustering algorithm for both shape variables and the PCA on allometry-free shape scores (**S5 File**).

### Mandible

**Fig 5:**
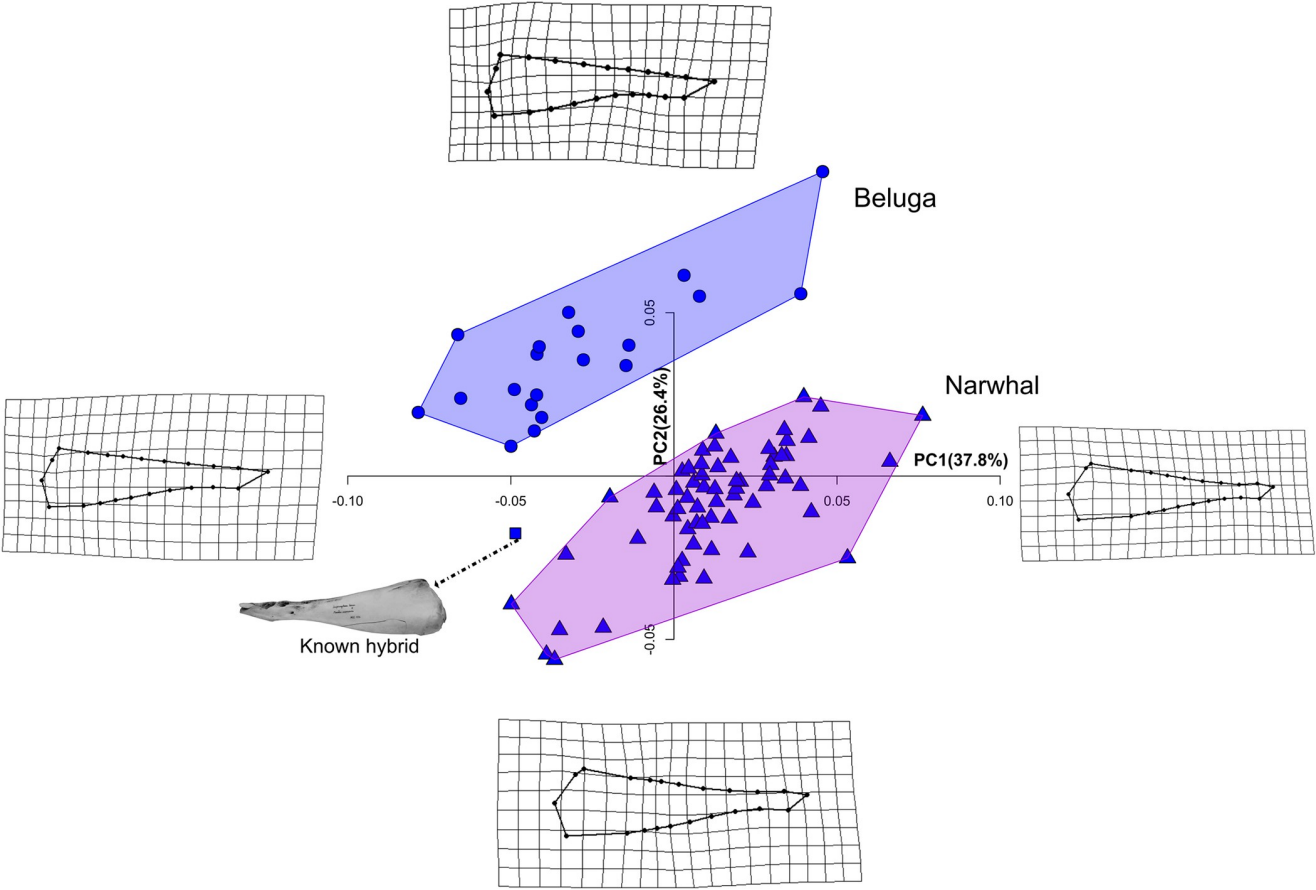
PCA on hemi-mandibles. Principal Component (PC) plot for the two-dimensional (2D) hemi-mandible monodontid dataset. Shape differences along the axis of the PC1 and PC2 are visualized with thin plate spline deformation grids. See text for details.

The first two PCs for the lingual view of the mandible accounted for 37.8% and 26.4% of the shape variation, respectively (**Fig 5; S6 File**), and showed a separation of the two species, while the hybrid occupies an intermediate position. Thin plate spline deformation grids showed that, for positive PC1 scores, mandibles are characterised by an enlargement of the mandibular foramen and a dorsoventrally lower mandible towards the tip. Negative PC1 scores are associated with anteroposterior compression of the mandibular foramen, a dorsoventrally higher mandible along the dental groove, a slight change on the posterior ventral tip of the angular process (LM 7), and on the dorsal tip of the coronoid process (LM 3). PC3 and PC4 explain 9.7% and 5.8% of the variation, respectively, and are not partitioned between species.

**Fig 6:**
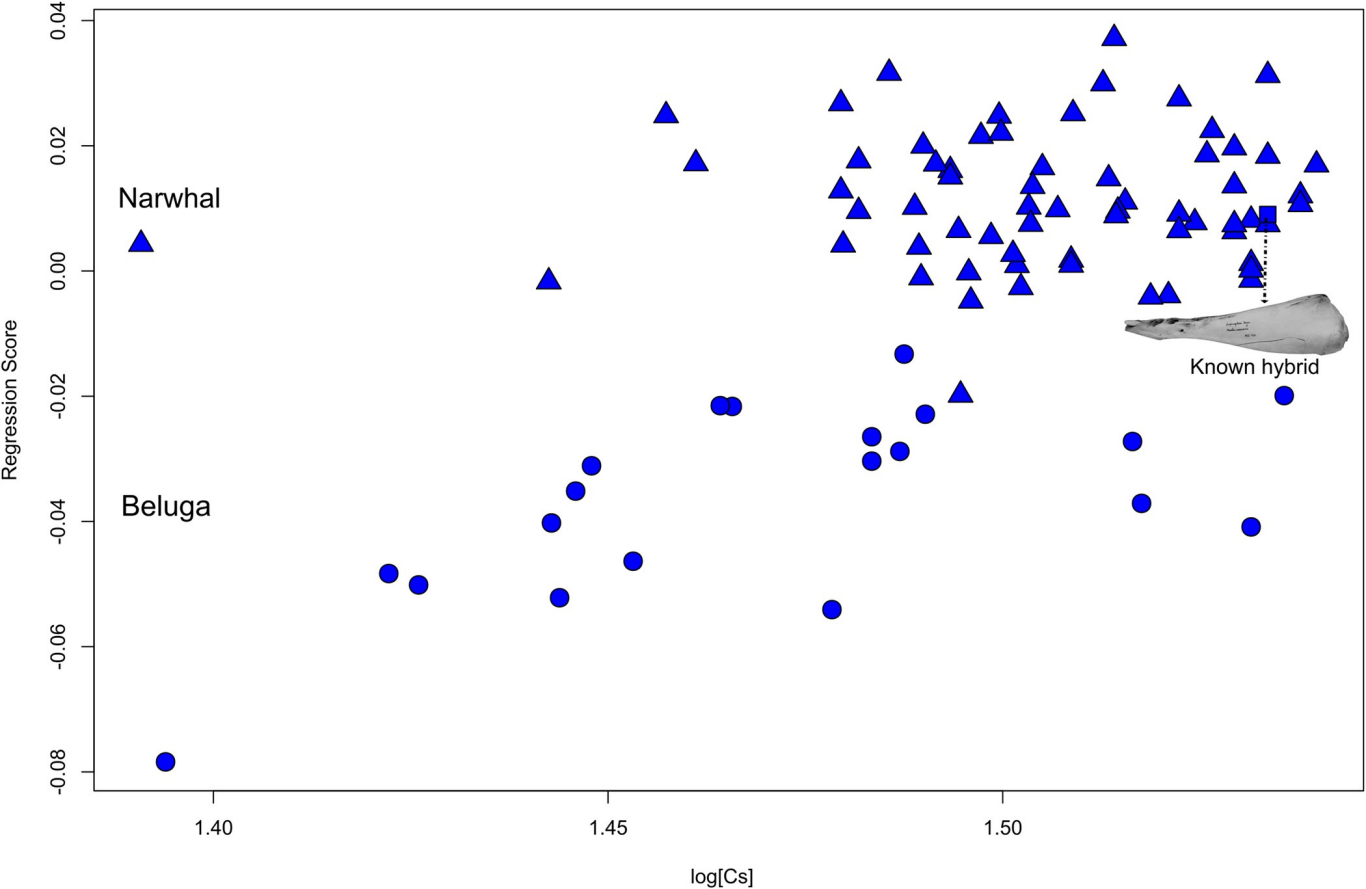
Allometry in mandible dataset. Regression of hemi-mandibular shape component against log CS for both narwhals (triangles) and belugas (circles) shows mandibular shape changes in relation to size. Dotted line shows the known hybrid hemi-mandible.

Regression of mandible shape against log CS (**Fig 6; S7 File**) for both species and the known hybrid reveals that size explains a significant component of variation (5.4%; P = 0.002). Further analyses could not be performed due to the small number of sexed individuals within each species.

#### Classification test based on the mandible

The UPGMA (**S4 Fig**) cluster analysis shows i) a slight overlap between narwhal and beluga, and ii) the known hybrid occupies an intermediate position, largely outside the narwhals and close to this overlap area, sharing similarities with both species. Cophenetic correlation for Procrustes coordinates is 0.7808. These results are confirmed by k-mean clustering which groups the known hybrid mandible with belugas. In contrast, using PCA-size free scores the hybrid mandible shape clusters within the narwhal group (**S5 File**).

### Skull integration

**Fig 7:**
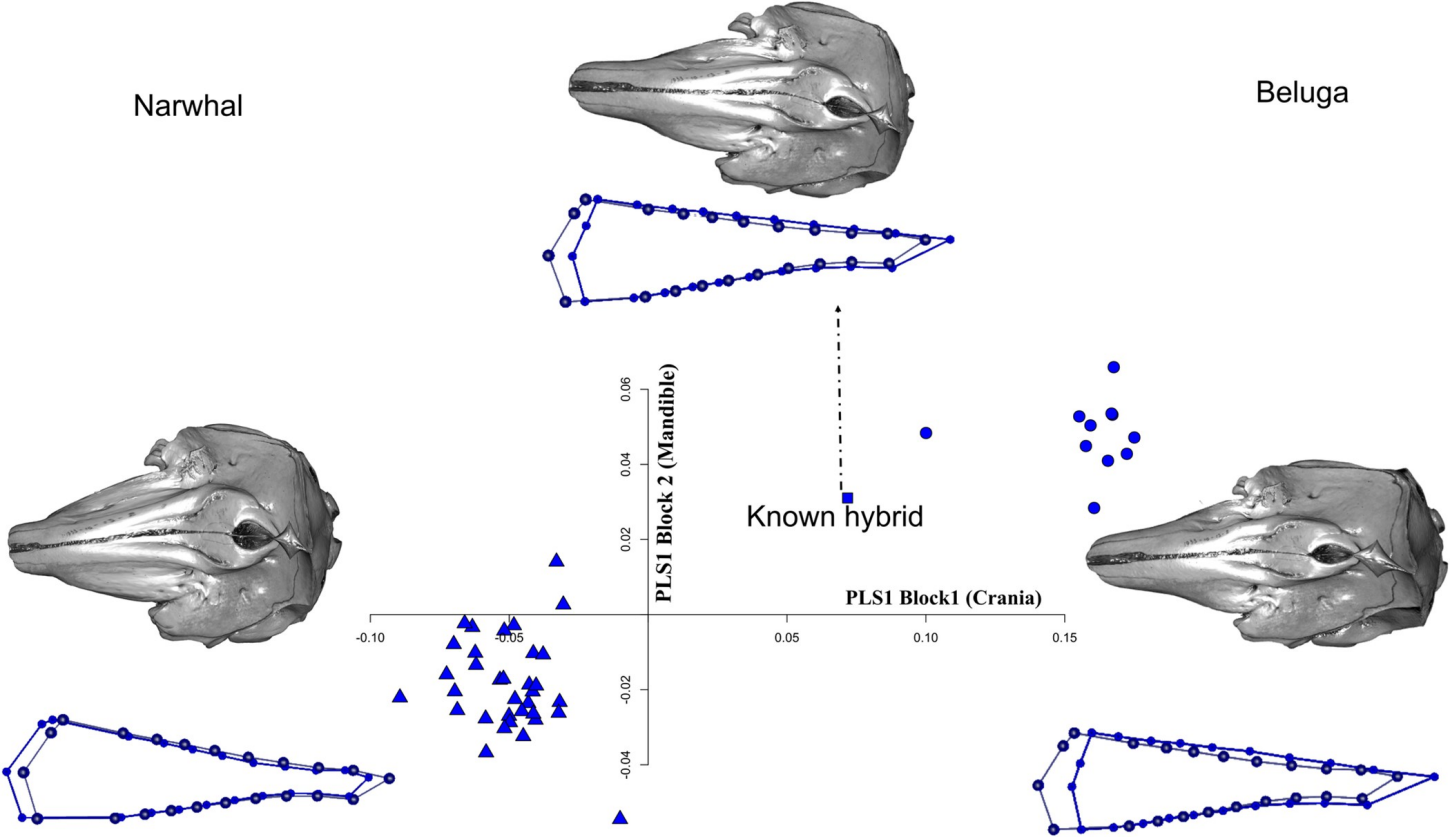
Skull integration. Scatter plot of the partial least square axis 1 (PLS1) of block1 (cranium) and block2 (mandible) of the Monodontidae. Shape differences can be viewed by 3D cranium warping and wireframe for the hemi-mandible; the light colour refers to the mean shape of the individuals, whereas the dark colour refers to extreme-most individual on the PLS1 axes. Correlation is 0.902. The dotted line indicates the hybrid warping and wireframe.

The 2B-PLS analysis is shown in **Fig 7**. The first pair of SAs account for 98.6% of the total squared covariance between cranium and mandible. The association between PLS1 scores of cranium and mandible was strong (*r* = 0.902) and significant (P < 0.0001). For negative PLS1 scores, narwhals showed a wider cranium and a shorter rostrum associated with a relatively shorter mandible and wider acoustic window, while for positive PLS1 scores belugas exhibited a narrower and more elongated cranium, and a relatively more elongated mandible with a wider body. The known hybrid morphology in the PLS morphospace clusters closer to belugas. Comparison of the cranium axis of narwhals and belugas (excluding the known hybrid), provided a PLS1 angle of 89.764° that supports the hypothesis of non-parallel integration trajectories (P = 0.9747). The same results were obtained for the mandible, with PLS1 showing an angle of 86.543° (P = 0.6472).

### Genomic analysis of the putative hybrid specimen

**Fig 8:**
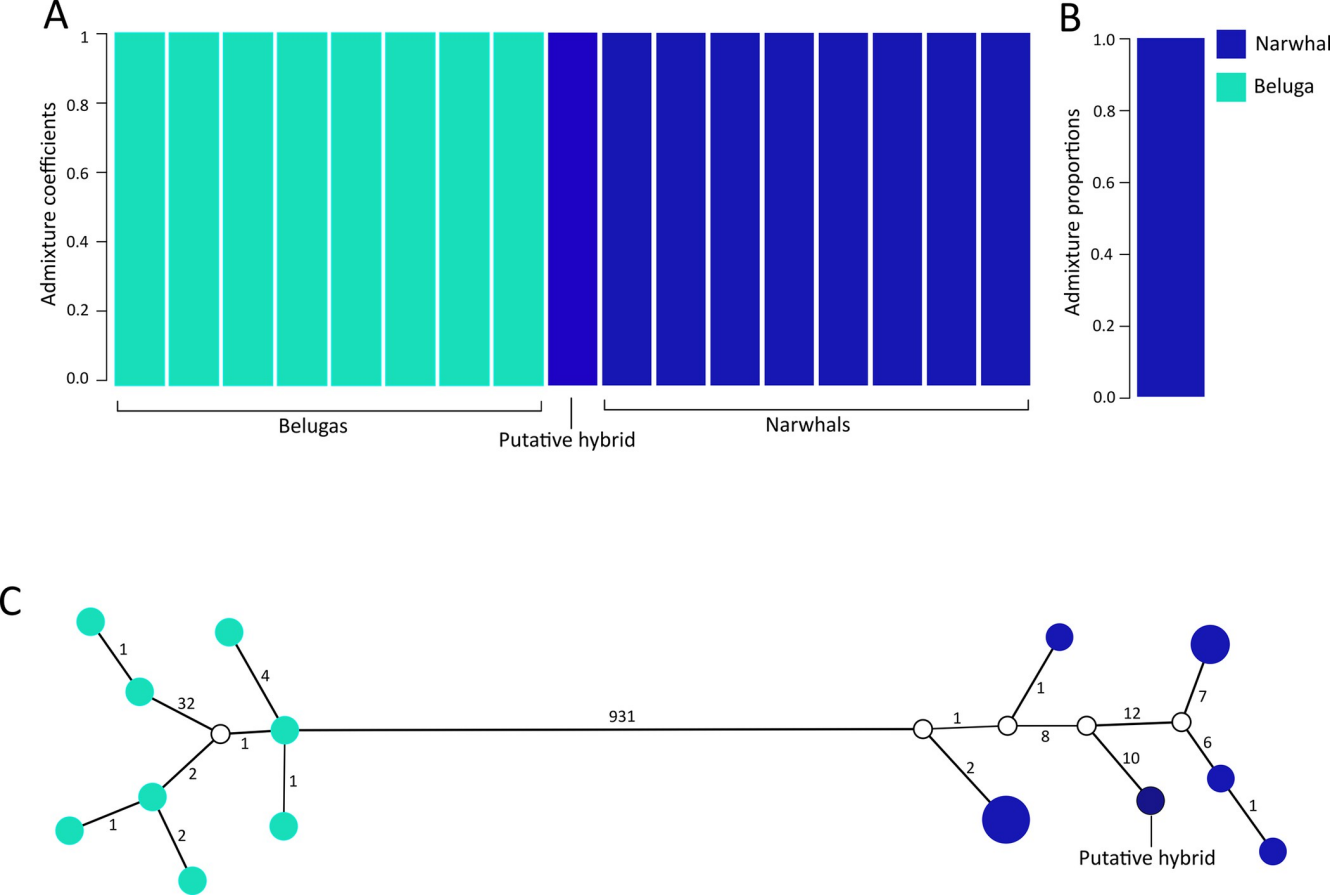
Ancestry analyses of NHMD 1963.44.1.4. (**A)** NGSadmix analysis of the putative hybrid and a reference panel of eight narwhal and eight beluga individuals. (**B)** fastNGSadmix analysis of the putative hybrid. (**C)** mitochondrial haplotype network. Circle size indicates relative number of individuals sharing a haplotype. Belugas are indicated in light blue, narwhals are indicated in dark blue. The putative hybrid (NHMD 1963.44.1.4) appears as a narwhal in both genomic and mtDNA analyses. White circles indicate intermediate haplotypes not found in the samples. Numbers indicate mutation steps between haplotypes; of note, distances between haplotypes are not to scale.

The genomic analyses showed that the putative hybrid was a male narwhal. In the NGSadmix analyses, the putative hybrid clustered with narwhals without any sign of admixture, a finding confirmed by the fastNGSadmix analyses (**Fig 8**). The PCA analyses based on the ‘complete’ and ‘fixed-sites’ datasets separated belugas and narwhals on PC1, capturing 68.3% and 91.3% of the variation, respectively (**S8 File**). For both data sets, the putative hybrid fell within the narwhal variation, supporting a non-admixed narwhal ancestry. The mitochondrial haplotype network showed that the putative hybrid clustered with the narwhal individuals, confirming the matriline of the specimen was narwhal (**Fig 8C**).

### Stable carbon and nitrogen isotope analysis

**Fig 9:**
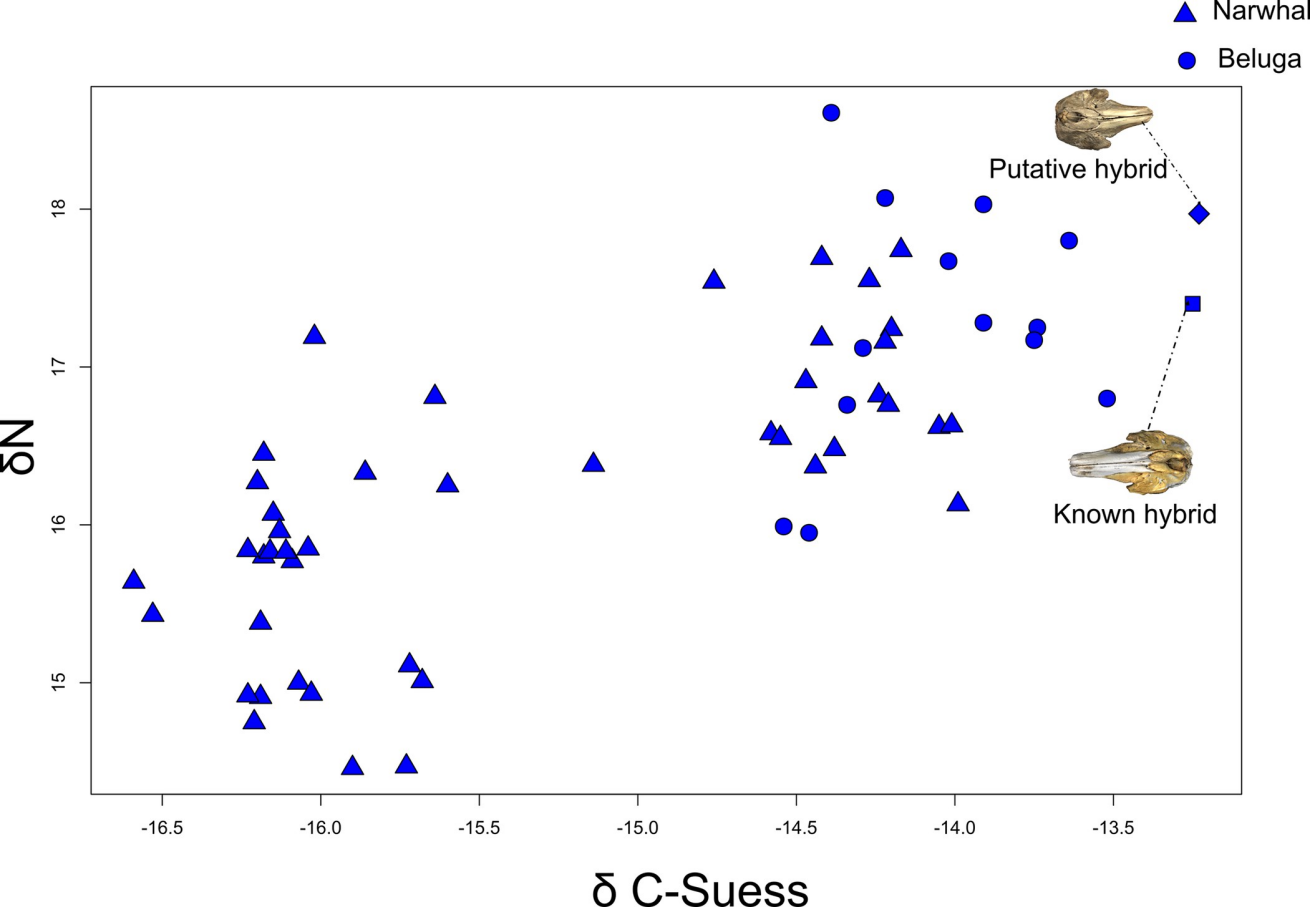
Stable carbon and nitrogen isotopic compositions in monodontids. Bone collagen stable isotopic composition of carbon (*δ*^13^C) and nitrogen (*δ*^15^N) of 45 narwhals (triangle), 13 belugas (circle), the known hybrid (square) and the putative hybrid (diamond).

Both the known and the putative hybrid had much higher *δ*^13^C values relative to narwhals and belugas. *δ*^15^N values of the known and putative hybrids were within the range observed in narwhal and beluga, although the putative hybrid showed a signal more similar to beluga. Statistical analyses support differences in mean *δ*^13^C between narwhals and belugas (t-test = 2.030, P < 0.0001) and in *δ*^15^N (t-test = 2.055, P = 0.0008) (**Fig 9**).

## Discussion

### Morphological description of the known hybrid

We present detailed morphological analyses of the skulls of the only known narwhal-beluga hybrid [5] and corresponding parental species. The hybrid displays an unusual head morphology with beluga cranium characters and a narwhal mandible. The narluga tooth count was the most important identifying feature, with five teeth in the upper row and four teeth in the bottom row per side. Teeth were apically worn testifying that they were in an erupted state and that the animal used them to catch its prey. A recently published study confirmed that the known hybrid male specimen analysed in this work resulted from hybridisation between a female narwhal and a male beluga [5].

We show that the cranial morphology of the hybrid resembles the beluga while the mandible is quite atypical because it presents all the characters of a narwhal, such as a relatively slimmer corpus and wider ramus (**Figs 6 and 7**). Such a mismatch in shape between cranium and mandible is also highlighted in the PLS morphospace where the hybrid clusters closely to the beluga but it is still an outlier. This suggests that hybrid morphology might be reflected differently in different parts of the skull (cranium versus mandible). It is also notable that patterns of morphological covariation within the hybrid skull are intermediate between the two parental species, with covariation between cranium and mandible in the hybrid most similar to belugas.

The relatively high hybrid *δ*^13^C value most closely resembles belugas, and is indicative of dietary similarities, while nitrogen values suggest trophic similarities with both parental species. The mandible in toothed whales plays an important role in feeding [68] and sound reception [69] due to the mandibular window which hosts an acoustically-linked fat pad that enables sound transmission to the middle ear. The more enlarged the mandibular foramen is, the more sensitive the individuals are to a greater peak frequency range, facilitating greater prey detection while deep-diving [70]. The relative bluntness of the jaw, or amblygnathy (amblos = blunt; gnathos = jaw), is also important in feeding. It can be represented by the mandibular width divided by the length, providing the mandibular bluntness index (MBI) [68]. Monodontids show a high MBI (very blunt mandible), and a decrease in the number of teeth (compared to other toothed whales species), which is strongly associated with suction feeding. In contrast, the hybrid shows an increased number of teeth. Moreover, our analyses also revealed that its size exceeds that of belugas and is similar to the larger narwhals, something that is also common in mammalian F1 hybrid skulls [71–74]. A larger body size can increase the ability to dive to greater depths, that could have also facilitated a deep diving and a bottom feeding strategy [5] in the known hybrid. Hence, size, tooth count, and enlargement of the area of the mandibular foramen in the mandible suggest closer resemblance to the narwhal and might be indicative of a deep diving bottom feeding strategy by the hybrid.

### Investigation of the putative hybrid

The putative hybrid specimen was found to be a narwhal with an upper row dental formula of 3–3, and two erupted teeth on each side. It is notable that narwhals have 6 pairs of dental papillae in the maxillae [2]. Occurrence of vestigial teeth has been noted in foetal specimens and in some cases persists in adults [75]. This specimen further confirms the recurring episode of male narwhals with a set of partly developed teeth other than the tusks.

In the case of the specimen studied here, dental papillae may have developed in teeth, erupted out of the gum, a feature that is regularly observed in odontocetes with a reduced dentition (e.g., the sperm whale *Physeter macrocephalus* on public view at the NHMD and several beaked whales [76, 77]). Our genetic and genomic analyses confirm that the putative hybrid is a mislabelled narwhal. The size and shape of the cranium of the putative hybrid also confirm that it is a narwhal (**Fig 4**). Its foraging habits resulted in higher *δ*^13^C values tentatively suggesting a more benthic diet compared to other narwhals. More specifically, it shows an unusually high carbon value relative to the other sampled narwhals with a nitrogen value within the range of those found in belugas but not in narwhals. Such a high value for carbon-13 is usually found in organisms having a more benthic diet [78]. We speculate that its unusual dental formula due to the presence of erupted teeth may have had an impact on its feeding ecology.

### Morphological differences between narwhals and belugas

The crania of the two species are clearly distinct. Both have elongated facial bones with the narwhal group having a less pointed and more robust rostrum with asymmetric premaxilla. The maxilla provides insertion for the upper row of teeth in belugas while some of the cranial differences in narwhals are explained by the development of the left maxillary tusks in males. The known hybrid is clearly intermediate in terms of the crania, while the putative hybrid occupies the narwhal morphospace.

Sexual shape dimorphism (excluding the presence/absence of the erupted left maxillary tusk in narwhals) was not detected for the crania of either species. This phenomenon has already been identified in different species of Odontoceti such as the false killer whale (*Pseudorca crassidens*), and the bottlenose dolphin (*Tursiops truncatus*) [79, 80]. The conservative social structure between sexes during foraging might be partially related with food sharing within populations [79, 81] and could explain why no SShD was detected.

### Sexual size dimorphism (SSD) in monodontids

Males are larger than females for narwhals only. This is probably due to the smaller beluga sexed sample size compared to the narwhal. Studies on narwhals [82], and in 5 stocks of belugas [81, 83, 84] in the Canadian Artic show larger males in both species. In particular, narwhal males display larger tusks, which correlate with testes mass [14] and reflect the males quality and fertility [85]. This would indicate the importance of the tusk in the mating system [83, 86], as a secondary sexual character that attracts females [14]. Both the known hybrid [5] and the putative hybrid were males. The known hybrid showed a relatively greater cranial size than that in all—male belugas but was similar to that in male narwhals. Size and shape of its mandible are compatible with those of male narwhals, again suggesting a mixed skull morphology for this hybrid specimen.

### Allometry

Allometry explains quite a significant portion of shape variation in the analysed samples, with size having a greater impact on skull shape in the beluga than the narwhal. We detected differing allometric slopes that can be explained by different growth processes between species. Belugas were expected to be of slightly larger size than narwhals due to more allometric growth, but this is not what we observed. Instead, cranial shape showed different allometric/intercept starting points and slopes between species indicating that belugas have a hyper allometric skull growth while this is hypo allometric in narwhals. Consequently, belugas should grow larger than narwhals, but a higher intercept starting point is expected in narwhals to compensate for their hypoallometric pattern. This also provides an indication that evolutionary forces have acted on different stages of cranial growth within the monodontid clade resulting in different allometric trajectories between the two species.

Between-sex differences in slopes were only found in narwhals which may be due to different energetic demands and investment by male narwhal as they grow their tusks, while females have a stronger maternal investment [33]. The different impact of size on shape might also be due to differences in adult growth and food partitioning between sexes [33]. In contrast, in belugas we found slopes did not differ between sexes, although as mentioned previously this might be due to a smaller number of sexed individuals. Conservation of the direction of shape changes with skull growth (between sexes) have been noted in false killer whales [79], and should be further investigated in beluga with a larger sexed sample.

### Skull integration

A strong correlation between cranium and mandible shape between and within species was found. Dissimilarities in the morphological integration between cranium and mandible across the two parental species were considerable. There was a clear separation between the two species and the hybrid, although the pattern of covariation placed the known hybrid close to the belugas (this seemed to largely reflect relative elongation of the rostrum and mandible, and the height of the braincase).

The pattern of cranial and mandibular covariation in toothed whales has rarely been explored within and between species [but see 52]. Although whales do not chew their food, functional covariation is expected to occur, otherwise occlusion of upper and lower jaw might be compromised. The differences in the cranio-mandibular complex between narwhal and beluga are quite evident with narwhals showing a wider cranium and mandible possibly due to the relative elongation of the rostrum and constraints imposed by the need for support the tusk. In contrast, the beluga possesses a relatively more elongated cranium and longer corpus of the mandible (in toothed whale defined as ending at the level of the last posterior alveolus [87]) and a shorter acoustic window implying differences in sound reception between species. These differences might be adaptive, being advantageous for a more benthic diet for the beluga. The dietary differences are supported by the isotopic analyses.

## Conclusions

We found that the known hybrid specimen displayed affinities with one parental type for the cranium but a different one for the mandible which could have led to misidentification. The putative hybrid specimen in this study was found to be a male narwhal that was mislabelled due to the presence of two erupted upper row teeth on both sides of the cranium and the lack of a maxillary left tusk. Finally, our analyses of allometry revealed interesting differences between belugas and narwhals which supported more detailed interspecific comparisons of growth in the future. In conclusion, geometric morphometrics is a useful tool to determine and identify hybrids and mislabelled specimens in museum collections. Our findings show how, for museum material, information on sample provenance and even the identified species, can be erroneous, and therefore showcase the utility of a combination of morphological, genetic and isotopic analyses.

## Supporting information

S1 Appendix List of specimens. (DOCX)

S1 Fig Map of Greenland. (TIF)

S2 Fig PCA on crania dataset with 42 landmarks. Principal Component (PC) plot of the cranial shape of the three-dimensional (3D) Monodontidae dataset with 42 landmarks. Shape differences along the axis of the PC1 and PC2 are visualized with warping in (A) dorsal, (B) ventral, (C) left lateral and (D) occipital view.
(TIF)

S3 Fig UPGMA cranium. UPGMA computed on the matrix of the Procrustes distances among the crania shape of the Monodontidae. Boot numbers indicates the reliability of groups. In this case 100% for narwhals (Mm), and 100% for belugas (Dl).
(TIF)

S4 Fig UPGMA mandible. UPGMA computed on the matrix of the Procrustes distances among the mandible shape of narwhal (Mm) and beluga (Dl) specimens.
(TIF)

S1 File 3D model of the known hybrid specimen (NHMD MCE 1356). (PLY)

S2 File DNA extraction procedure. (DOCX)

S3 File Raw data of the cranium of the 157 monodontids. (TXT)

S4 File Cranial allometry. Procrustes ANOVA to test for allometry in (A) 49 sexed narwhals and (B) 24 sexed belugas. Significance is highlighted in bold.
(DOCX)

S5 File K-mean clustering. Cluster-classifications from k-means clustering based on cranial (A) and mandibular (B) shape variables and size free shape variables of narwhals (Mm) and belugas (Dl). Cluster 1 was assigned to Dl while the cluster 2 to Mm. The known and putative hybrids are highlighted in bold.
(XLSX)

S6 File Raw data of the 2D hemi mandible of 85 monodontids. (TPS)

S7 File Centroid size of the 2D hemi mandible of the 85 monodontids. (TXT)

S8 File PCA on genetic dataset. PCAngsd analysis of the (A) complete dataset and (B) Fixed-Sites Dataset. In A and B, the proportion of genetic variance captured by each component is indicated in parentheses.
(TIF)
