# Supplementary material for: Skull ecomorphological variation of narwhals (Monodon monoceros, Linnaeus 1758) and belugas (Delphinapterus leucas, Pallas 1776) reveals phenotype of their hybrids
Source: PLoS One. 2022 Aug 12;17(8):e0273122. doi: 10.1371/journal.pone.0273122 (PMC9374245; doi:10.1371/journal.pone.0273122)
Supplement: S1 Appendix — (DOCX) [file pone.0273122.s001.docx]

**S1 Appendix- List of Monodontidae specimens**

| **Genus** | **Species** | **Museum** | **ID** |
| --- | --- | --- | --- |
| *Monoceros* | *monoceros* | NHMD | 1358.493 |
| *Monoceros* | *monoceros* | NHMD | 1361.537 |
| *Monoceros* | *monoceros* | NHMD | 1364.655 |
| *Monoceros* | *monoceros* | NHMD | 1365.656 |
| *Monoceros* | *monoceros* | NHMD | 1366.654 |
| *Monoceros* | *monoceros* | NHMD | 1370.3 |
| *Monoceros* | *monoceros* | NHMD | 1371.843 |
| *Monoceros* | *monoceros* | NHMD | 1372.5 |
| *Monoceros* | *monoceros* | NHMD | 1380 |
| *Monoceros* | *monoceros* | NHMD | 1386.853 |
| *Monoceros* | *monoceros* | NHMD | 1388.21 |
| *Monoceros* | *monoceros* | NHMD | 1389.22 |
| *Monoceros* | *monoceros* | NHMD | 1392.864.25 |
| *Monoceros* | *monoceros* | NHMD | 1393.26 |
| *Monoceros* | *monoceros* | NHMD | 1406 |
| *Monoceros* | *monoceros* | NHMD | 1408 |
| *Monoceros* | *monoceros* | NHMD | 1409.845 |
| *Monoceros* | *monoceros* | NHMD | 1432.261 |
| *Monoceros* | *monoceros* | NHMD | 1433.262 |
| *Monoceros* | *monoceros* | NHMD | 1434.261 |
| *Monoceros* | *monoceros* | NHMD | 1435.262 |
| *Monoceros* | *monoceros* | NHMD | 1437.262 |
| *Monoceros* | *monoceros* | NHMD | 1438.262 |
| *Monoceros* | *monoceros* | NHMD | 1439.262 |
| *Monoceros* | *monoceros* | NHMD | 1440.263 |
| *Monoceros* | *monoceros* | NHMD | 1441.263 |
| *Monoceros* | *monoceros* | NHMD | 1442.263 |
| *Monoceros* | *monoceros* | NHMD | 1445.275 |
| *Monoceros* | *monoceros* | NHMD | 1451.276 |
| *Monoceros* | *monoceros* | NHMD | 1452.276 |
| *Monoceros* | *monoceros* | NHMD | 1454 |
| *Monoceros* | *monoceros* | NHMD | 1455 |
| *Monoceros* | *monoceros* | NHMD | 1457.277 |
| *Monoceros* | *monoceros* | NHMD | 1458.277 |
| *Monoceros* | *monoceros* | NHMD | 1459.277 |
| *Monoceros* | *monoceros* | NHMD | 1460.277 |
| *Monoceros* | *monoceros* | NHMD | 1461 |
| *Monoceros* | *monoceros* | NHMD | 1462.277 |
| *Monoceros* | *monoceros* | NHMD | 1463.277 |
| *Monoceros* | *monoceros* | NHMD | 1574 |
| *Monoceros* | *monoceros* | NHMD | 1578 |
| *Monoceros* | *monoceros* | NHMD | 1579.2 |
| *Monoceros* | *monoceros* | NHMD | 1580 |
| *Monoceros* | *monoceros* | NHMD | 1584-4107 |
| *Monoceros* | *monoceros* | NHMD | 1456.277 |
| *Delphinapterus* | *leucas* | NHMD | 1312 |
| *Delphinapterus* | *leucas* | NHMD | 1313 |
| *Delphinapterus* | *leucas* | NHMD | 1318 |
| *Delphinapterus* | *leucas* | NHMD | 1319 |
| *Delphinapterus* | *leucas* | NHMD | 1320 |
| *Delphinapterus* | *leucas* | NHMD | 1321 |
| *Delphinapterus* | *leucas* | NHMD | 1322 |
| *Delphinapterus* | *leucas* | NHMD | 1323 |
| *Delphinapterus* | *leucas* | NHMD | 1329 |
| *Delphinapterus* | *leucas* | NHMD | 1334 |
| *Delphinapterus* | *leucas* | NHMD | 1335 |
| *Delphinapterus* | *leucas* | NHMD | 1338 |
| *Delphinapterus* | *leucas* | NHMD | 1343 |
| *Delphinapterus* | *leucas* | NHMD | 1344 |
| *Delphinapterus* | *leucas* | NHMD | 1350 |
| *Delphinapterus* | *leucas* | NHMD | 1351 |
| *Monoceros* | *monoceros* | NHMD | 12x |
| *Monoceros* | *monoceros* | NHMD | 1373.6 |
| *Monoceros* | *monoceros* | NHMD | 1378.85 |
| *Monoceros* | *monoceros* | NHMD | 1385.18 |
| *Monoceros* | *monoceros* | NHMD | 1387.859.20 |
| *Monoceros* | *monoceros* | NHMD | 13x |
| *Monoceros* | *monoceros* | NHMD | 1410 |
| *Monoceros* | *monoceros* | NHMD | 1416 |
| *Monoceros* | *monoceros* | NHMD | 1422 |
| *Monoceros* | *monoceros* | NHMD | 1423 |
| *Monoceros* | *monoceros* | NHMD | 1427 |
| *Monoceros* | *monoceros* | NHMD | 1428 |
| *Monoceros* | *monoceros* | NHMD | 1464.278 |
| *Monoceros* | *monoceros* | NHMD | 14x |
| *Monoceros* | *monoceros* | NHMD | 1583.411 |
| *Monoceros* | *monoceros* | NHMD | 1592M |
| *Monoceros* | *monoceros* | NHMD | 1593.412 |
| *Monoceros* | *monoceros* | NHMD | 15x |
| *Monoceros* | *monoceros* | NHMD | 17x |
| *Monoceros* | *monoceros* | NHMD | 20 |
| *Monoceros* | *monoceros* | NHMD | 2629.144 |
| *Monoceros* | *monoceros* | NHMD | 2751.145 |
| *Monoceros* | *monoceros* | NHMD | 2757 |
| *Monoceros* | *monoceros* | NHMD | 43 |
| *Monoceros* | *monoceros* | NHMD | 47 |
| *Monoceros* | *monoceros* | NHMD | 56x |
| *Monoceros* | *monoceros* | NHMD | 5x |
| *Monoceros* | *monoceros* | NHMD | 84 |
| *Monoceros* | *monoceros* | NHMD | 847.1411 |
| *Monoceros* | *monoceros* | NHMD | 85 |
| *Monoceros* | *monoceros* | NHMD | 888 |
| *Monoceros* | *monoceros* | NHMD | 950 |
| *Monoceros* | *monoceros* | NHMD | 951 |
| *Monoceros* | *monoceros* | NHMD | 952 |
| *Monoceros* | *monoceros* | NHMD | 953 |
| *Monoceros* | *monoceros* | NHMD | 956 |
| *Monoceros* | *monoceros* | NHMD | 974 |
| *Monoceros* | *monoceros* | NHMD | 9x |
| *Monoceros* | *monoceros* | MNHN | 1869.759 |
| *Monoceros* | *monoceros* | MNHN | 1903.103 |
| *Monoceros* | *monoceros* | NMS | 1895.30.3 |
| *Delphinapterus* | *leucas* | NHMD | 85129 |
| *Delphinapterus* | *leucas* | NHMD | 85130 |
| *Delphinapterus* | *leucas* | NHMD | 1.17 |
| *Delphinapterus* | *leucas* | NHMD | 1.4.1963.37.1854 |
| *Delphinapterus* | *leucas* | NHMD | 1.4.1963.42.1854 |
| *Delphinapterus* | *leucas* | NHMD | 10 |
| *Delphinapterus* | *leucas* | NHMD | 11 |
| *Delphinapterus* | *leucas* | NHMD | 12 |
| *Delphinapterus* | *leucas* | NHMD | 1306 |
| *Delphinapterus* | *leucas* | NHMD | 1311 |
| *Delphinapterus* | *leucas* | NHMD | 1346 |
| *Delphinapterus* | *leucas* | NHMD | 1346 |
| *Delphinapterus* | *leucas* | NHMD | 1347 |
| *Delphinapterus* | *leucas* | NHMD | 1352 |
| *Delphinapterus* | *leucas* | NHMD | 1353 |
| *Delphinapterus* | *leucas* | NHMD | 1355 |
| *Known* | *hybrid* | NHMD | 1356 |
| *Delphinapterus* | *leucas* | NHMD | 279 |
| *Delphinapterus* | *leucas* | NHMD | 3.968 |
| *Delphinapterus* | *leucas* | NHMD | 34.15.3.1878 |
| *Delphinapterus* | *leucas* | NHMD | 350 |
| *Delphinapterus* | *leucas* | NHMD | 38.1854/1.4.1963 |
| *Delphinapterus* | *leucas* | NHMD | 39.1.4.1965.1854 |
| *Delphinapterus* | *leucas* | NHMD | 4.3.1870 |
| *Delphinapterus* | *leucas* | NHMD | 40.1.4.1963.1854 |
| *Delphinapterus* | *leucas* | NHMD | 41.1.4.1963.1854 |
| *Delphinapterus* | *leucas* | NHMD | 43.1.4.63.1854 |
| *Delphinapterus* | *leucas* | NHMD | 43.14.12.1893 |
| *Delphinapterus* | *leucas* | NHMD | 44.7.8.1905 |
| *Putative* | *hybrid* | NHMD | 1963.44.1.4 |
| *Delphinapterus* | *leucas* | NHMD | 45 |
| *Delphinapterus* | *leucas* | NHMD | 47 |
| *Delphinapterus* | *leucas* | NHMD | 50 |
| *Delphinapterus* | *leucas* | NHMD | 51 |
| *Delphinapterus* | *leucas* | NHMD | 52 |
| *Delphinapterus* | *leucas* | NHMD | 54 |
| *Delphinapterus* | *leucas* | NHMD | 55 |
| *Delphinapterus* | *leucas* | NHMD | 56 |
| *Delphinapterus* | *leucas* | NHMD | 57 |
| *Delphinapterus* | *leucas* | NHMD | 58 |
| *Delphinapterus* | *leucas* | NHMD | 59 |
| *Delphinapterus* | *leucas* | NHMD | 60 |
| *Delphinapterus* | *leucas* | NHMD | 61 |
| *Delphinapterus* | *leucas* | NHMD | 63 |
| *Delphinapterus* | *leucas* | NHMD | 9 |
| *Delphinapterus* | *leucas* | MNHN | 1894.328 |
| *Delphinapterus* | *leucas* | MNHN | 1928.196 |
| *Delphinapterus* | *leucas* | MNHN | 1928.197 |
| *Delphinapterus* | *leucas* | MNHN | 1971.156 |
| *Delphinapterus* | *leucas* | MNHN | 1885.606 |
| *Delphinapterus* | *leucas* | MNHN | 1894.325 |
| *Delphinapterus* | *leucas* | MNHN | 1901.48 |
| *Delphinapterus* | *leucas* | NMS | 1876.12.1 |
| *Delphinapterus* | *leucas* | NMS | 2014.9 |
| *Delphinapterus* | *leucas* | LaS | 868 |
