# Supplementary figures and images for: Skull ecomorphological variation of narwhals (Monodon monoceros, Linnaeus 1758) and belugas (Delphinapterus leucas, Pallas 1776) reveals phenotype of their hybrids
Source: PLoS One. 2022 Aug 12;17(8):e0273122. doi: 10.1371/journal.pone.0273122 (PMC9374245; doi:10.1371/journal.pone.0273122)

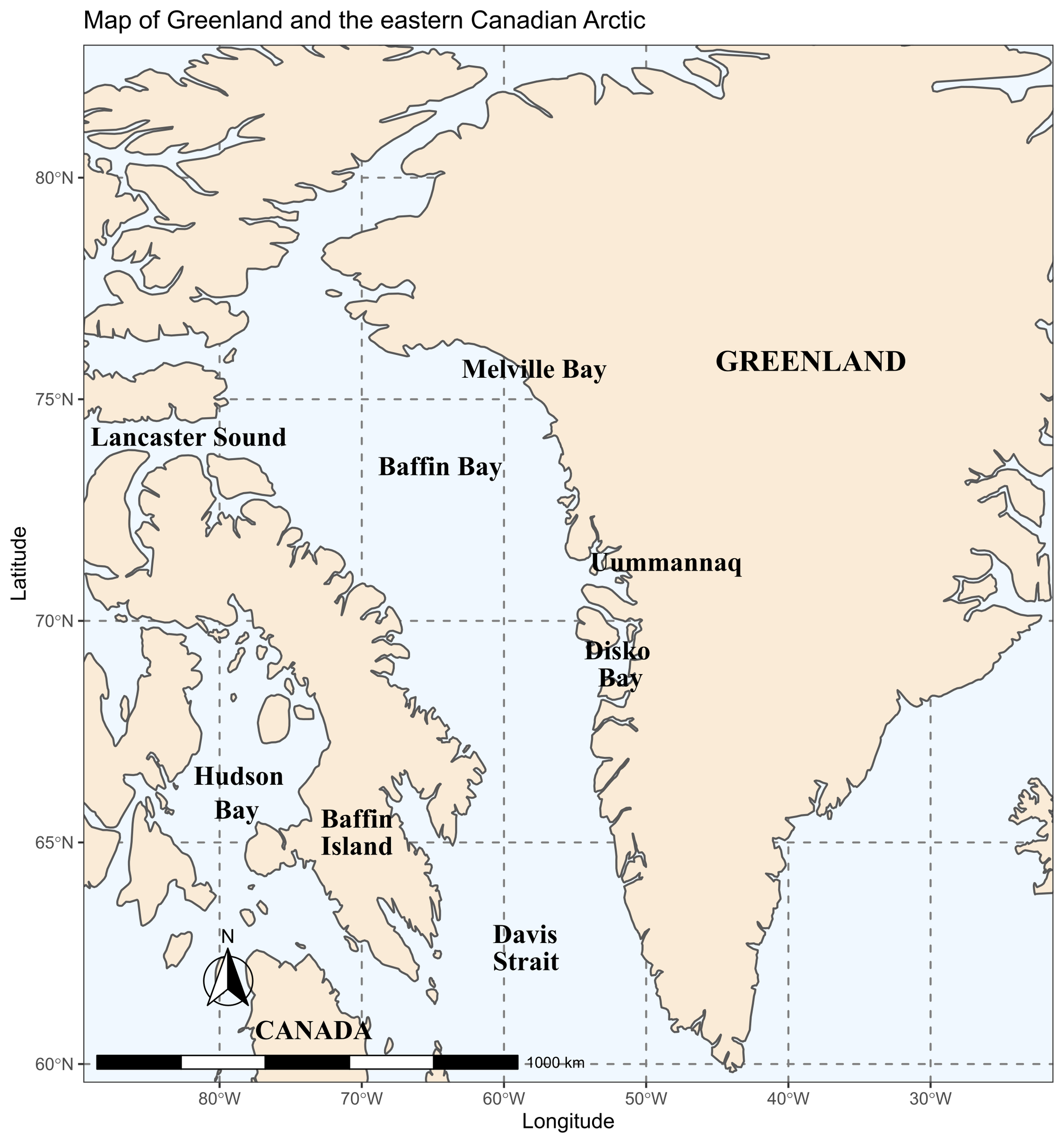

Supplement: S1 Fig — (TIF) [file pone.0273122.s002.tif]

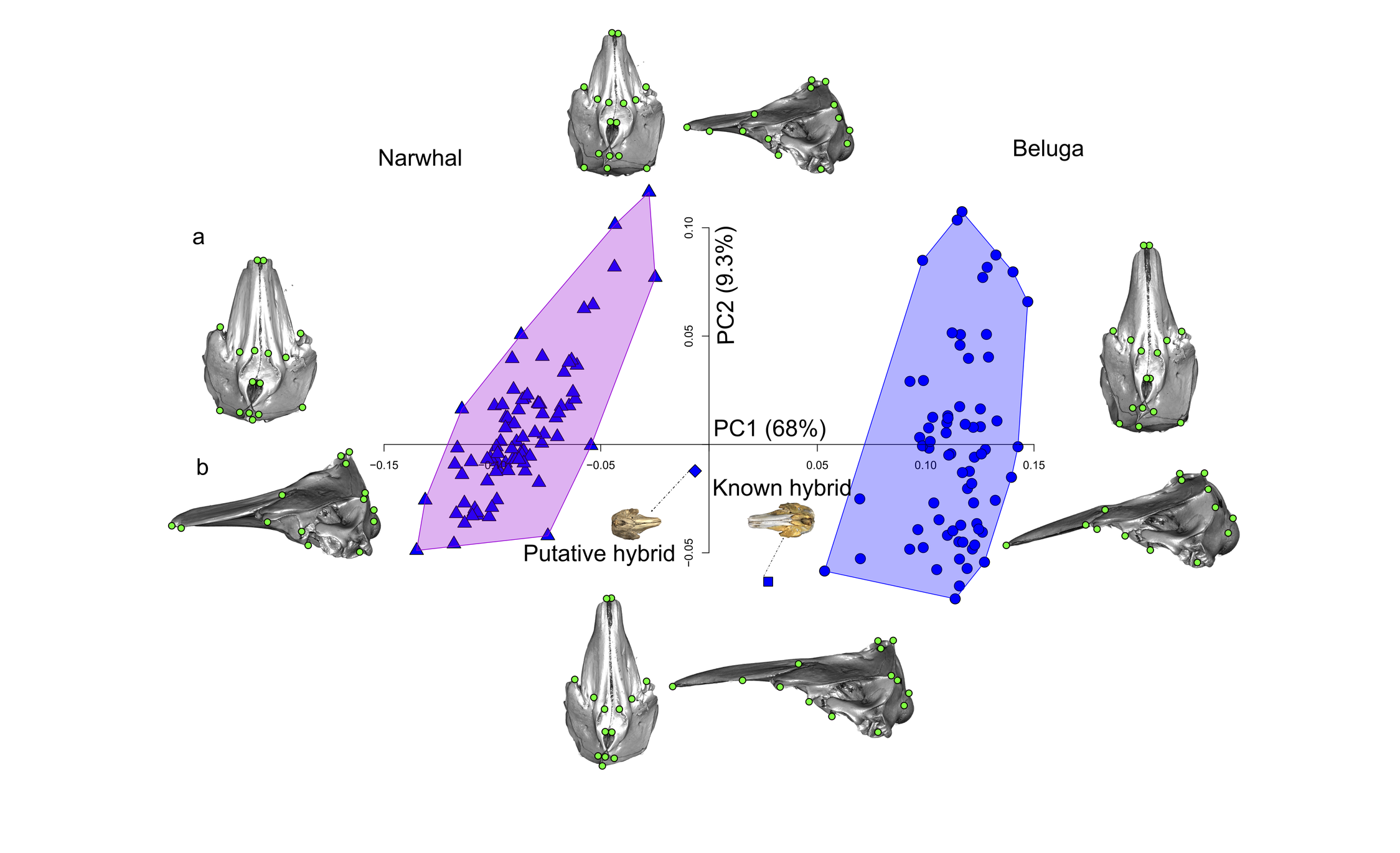

Supplement: S2 Fig — Principal Component (PC) plot of the cranial shape of the three-dimensional (3D) Monodontidae dataset with 42 landmarks. Shape differences along the axis of the PC1 and PC2 are visualized with warping in (A) dorsal, (B) ventral, (C) left lateral and (D) occipital view. (TIF) [file pone.0273122.s003.tif]

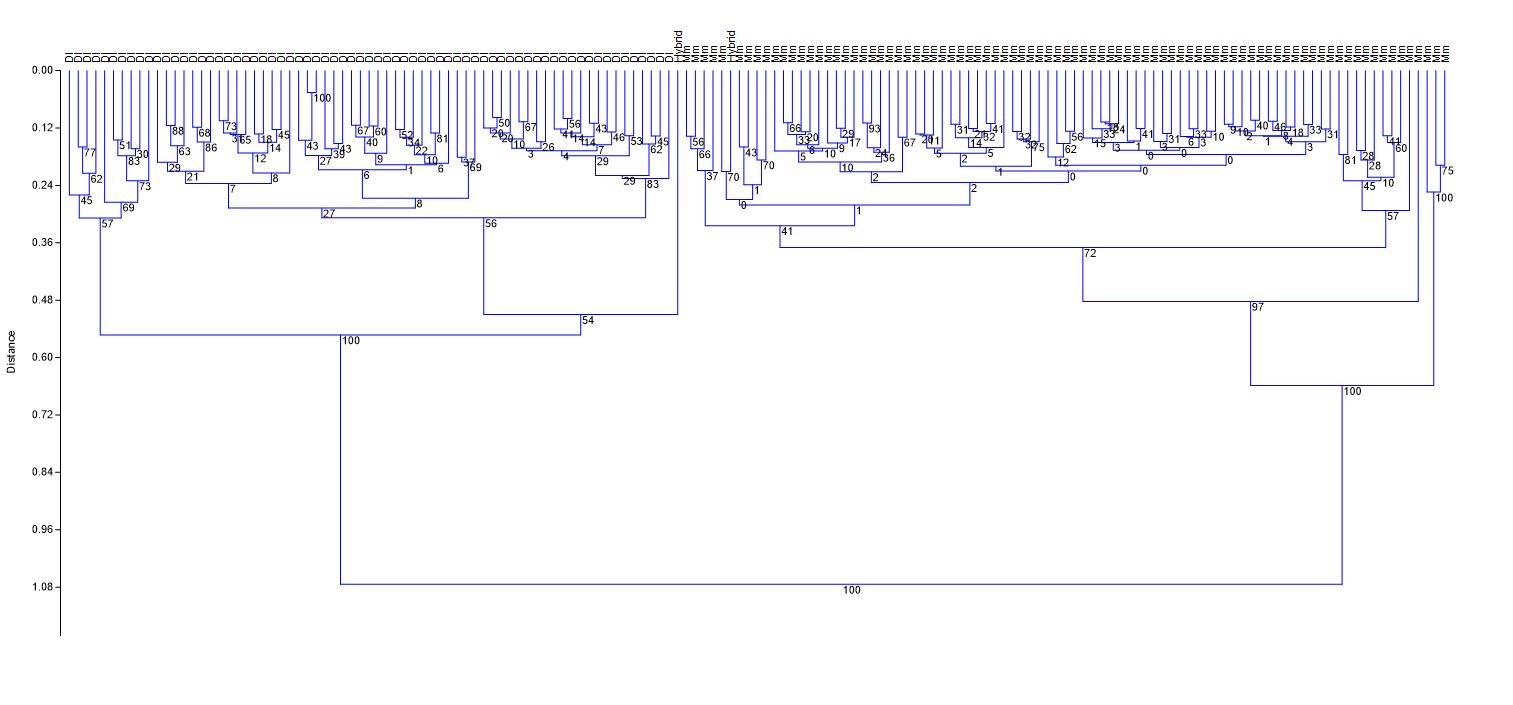

Supplement: S3 Fig — UPGMA computed on the matrix of the Procrustes distances among the crania shape of the Monodontidae. Boot numbers indicates the reliability of groups. In this case 100% for narwhals (Mm), and 100% for belugas (Dl). (TIF) [file pone.0273122.s004.tif]

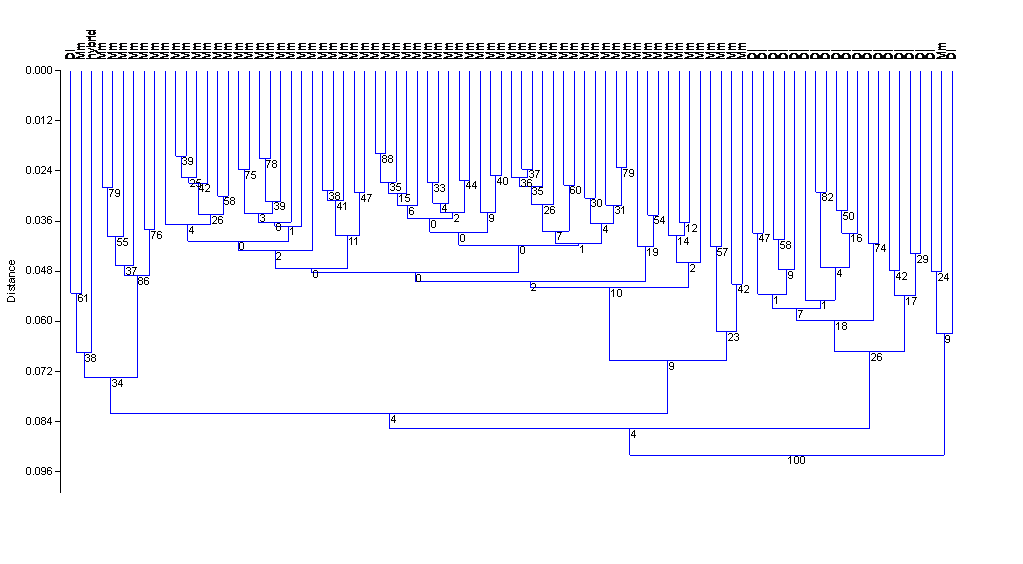

Supplement: S4 Fig — UPGMA computed on the matrix of the Procrustes distances among the mandible shape of narwhal (Mm) and beluga (Dl) specimens. (TIF) [file pone.0273122.s005.tif]

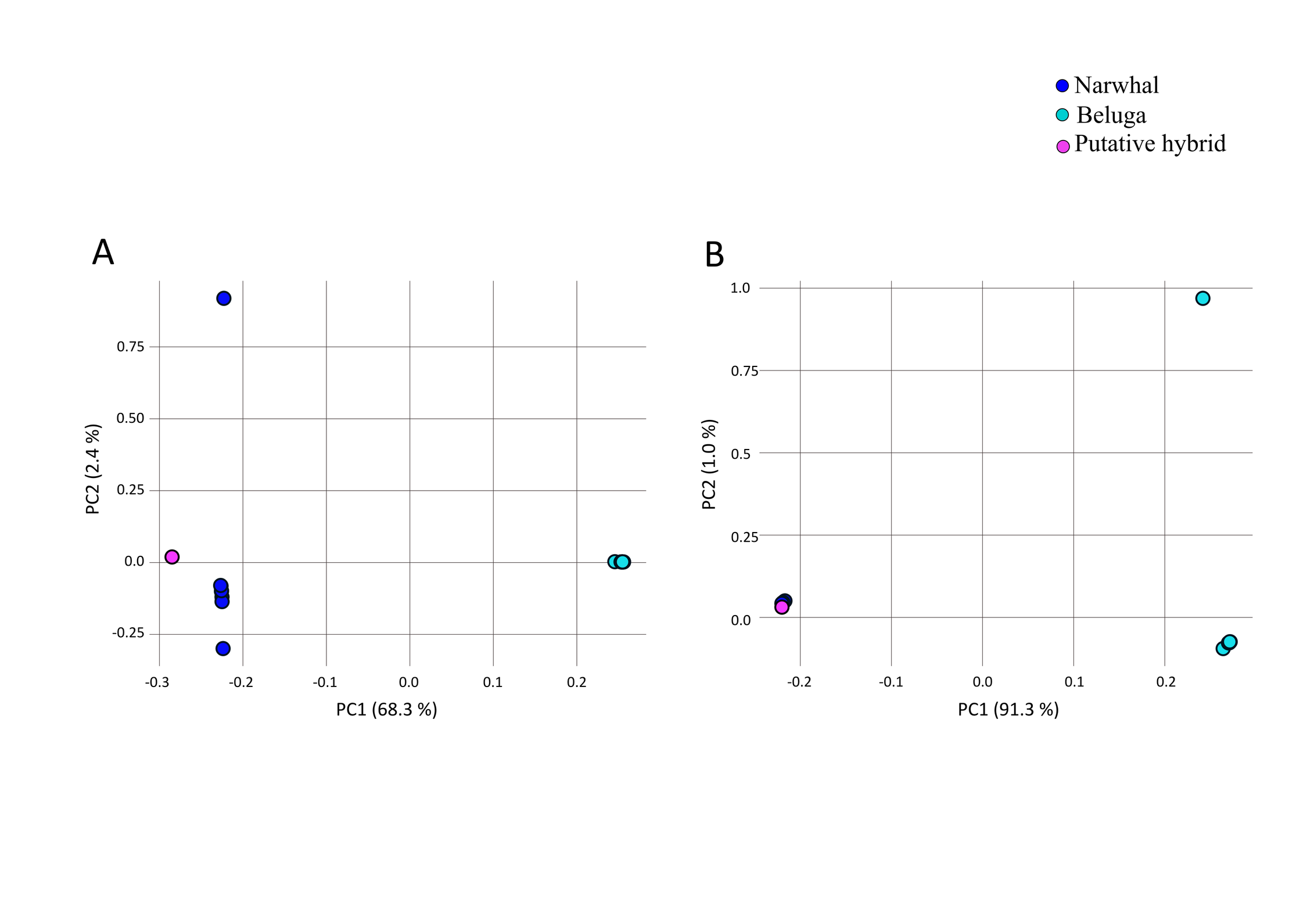

Supplement: S8 File — PCAngsd analysis of the (A) complete dataset and (B) Fixed-Sites Dataset. In A and B, the proportion of genetic variance captured by each component is indicated in parentheses. (TIF) [file pone.0273122.s013.tif]
