# Supplementary material for: Skull ecomorphological variation of narwhals (Monodon monoceros, Linnaeus 1758) and belugas (Delphinapterus leucas, Pallas 1776) reveals phenotype of their hybrids
Source: PLoS One. 2022 Aug 12;17(8):e0273122. doi: 10.1371/journal.pone.0273122 (PMC9374245; doi:10.1371/journal.pone.0273122)
Supplement: S2 File — (DOCX) [file pone.0273122.s007.docx]

## Methods

DNA extraction

To identify the genetic ancestry of the putative hybrid, we generated genome-wide genetic data and compared it to a reference panel of eight belugas and eight narwhals, following the methodology applied by Skovrind et al. [(2019)](https://paperpile.com/c/5l1Ezu/PJ1W/?noauthor=1). Using a hand-held Dremel, 50 mg of bone powder was drilled from the specimen. Genomic DNA was extracted from the bone powder in a dedicated ancient DNA laboratory at the University of Copenhagen using the extraction buffer described by Dabney et al [(2013)](https://paperpile.com/c/5l1Ezu/fwyv/?noauthor=1) with an additional pre-digest stage of 30 min as recommended by Damgaard et al [(Damgaard et al. 2015)](https://paperpile.com/c/5l1Ezu/tQeg). The extraction was concentrated by first using Amicon Ultra 30 kDa Centrifugal Filter Units and then further concentrated and cleaned using Qiagen Minelute tubes. The extract was built into Illumina libraries as described in Meyer and Kircher [(2010)](https://paperpile.com/c/5l1Ezu/OXXO/?noauthor=1). We used qPCR to quality check the libraries and calculate the appropriate number of PCR cycles required to sufficiently amplify them without causing overamplification. The libraries were then sequenced on the Illumina HiSeq 2500 platform using 80 bp SE technology. Raw sequencing data from eight belugas and eight was downloaded from the Electronic Research Data Archive (ERDA) <http://www.erda.dk/public/archives/YXJjaGl2ZS16NXRqTXE=/published-archive.html>.

Data processing

Initial data processing was performed within Paleomix v1.3.2 [(Schubert et al. 2014)](https://paperpile.com/c/5l1Ezu/6Shs). Sequencing adapters were removed from the raw sequencing reads using AdapterRemoval v2.3.1 [(Lindgreen 2012)](https://paperpile.com/c/5l1Ezu/kZDN) applying a minimum read length of 25 bp. Reads were mapped to the nuclear orca genome (*Orcinus orca*, Genbank accession: GCA_000331955.2) using the BWA Backtrack algorithm [(H. Li and Durbin 2009)](https://paperpile.com/c/5l1Ezu/s83N) while disabling the seed length and excluding repeat regions available from Genbank. Reads which mapped to multiple locations were removed using the MarkDuplicates from Picard [(Broad Institute 2016)](https://paperpile.com/c/5l1Ezu/Lr7C) and reads with read quality or mapping quality scores below 25 were removed using SAMtools v1.9 [(Heng Li et al. 2009)](https://paperpile.com/c/5l1Ezu/mlE3). The final alignment was realigned around indels using GATK [(McKenna et al. 2010)](https://paperpile.com/c/5l1Ezu/2nmN). The belugas and narwhals in the reference panel were also mapped to their respective mitochondrial genomes (Genbank accessions: KY88849.1) while the putative hybrid were mapped to the mitochondrial genomes of both species.

Data filtering

The belugas and narwhals in the reference panel had an average nuclear genome-wide coverage of 0.25x and 0.19x, respectively, and the putative hybrid had a nuclear coverage of 0.93x. Using ANGSD v0.931 [(Korneliussen, Albrechtsen, and Nielsen 2014)](https://paperpile.com/c/5l1Ezu/MNSi) we estimated the genotype likelihoods with the GATK method [(McKenna et al. 2010)](https://paperpile.com/c/5l1Ezu/2nmN), excluded non variable sites (*p* value < 1e^-4^), triallelic sites and sites with a global read depth below 3 and above 10. This dataset (Complete Dataset) included 4,281,320 sites. We further filtered the data to only include sites with (i) three or more reads from unique belugas, (ii) three or more reads from unique narwhals and (iii) one to four reads in the putative hybrid and (iv) belugas and narwhals in the reference panel were fixed for alternate alleles. This dataset (Fixed-Sites Dataset) included 75,362 sites. All subsequent nuclear analyses were performed on both datasets. Two mitochondrial alignments were constructed using using MAFFT v7.392, including the 16 reference panel samples and either the putative hybrid mapped to the beluga mitochondrial genome or the putative hybrid mapped to the narwhal mitochondrial genome.

Ancestry analyses

We estimated the individual admixture coefficients of the putative hybrid and the belugas and narwhals in the reference panel with NGSadmix [(Skotte, Korneliussen, and Albrechtsen 2013)](https://paperpile.com/c/5l1Ezu/9HMx), while specifying two populations (K = 2) and a minimum minor allele frequency of 0.1. We performed one hundred runs and the run with the highest likelihood was used for subsequent interpretation. We also estimated the admixture proportions of the putative hybrid using fastNGSadmix [(Jørsboe, Hanghøj, and Albrechtsen 2017)](https://paperpile.com/c/5l1Ezu/fSBO), applying 100 bootstraps. FastNGSadmix uses allele frequencies of species or populations in a reference panel and the genotype likelihoods of a single individual to estimate its admixture proportions. The allele frequencies of the sites included in the Complete and the Fixed-Sites Datasets were estimated for the belugas and narwhals using NGSadmix as described on the fastNGSadmix website <https://tinyurl.com/yc8bytwr>. To further visualise the ancestry of the putative hybrid we estimated a covariance matrix and performed a principal component analysis (PCA) using PCAngsd v0.95 [(Meisner and Albrechtsen 2018)](https://paperpile.com/c/5l1Ezu/RWIH) with default settings, except the minimum minor allele frequency, which was set to 0.1.

The mean mitochondrial coverage of the putative hybrid was substantially higher (158.6x) when mapped to the narwhal mitogenome than when mapped to the beluga mitogenome (75.5x). Belugas and narwhals in the reference panel had mean mitochondrial coverages of 78.3x and 84.6x, respectively. Mitochondrial fasta sequences were extracted using ANGSD, specifying a consensus base and a minimum read depth of five. The two mitochondrial alignments were used to construct median-joining haplotype networks [(Bandelt et al. 1999)](https://paperpile.com/c/5l1Ezu/zoZz) with popART v.1.7 [(Leigh and Bryant 2015)](https://paperpile.com/c/5l1Ezu/NnVw) excluding sites with missing data.

References

[Broad Institute. 2016. “Picard Toolkit.” GitHub Repository. 2016.](http://paperpile.com/b/5l1Ezu/Lr7C) <http://broadinstitute.github.io/picard/>[.](http://paperpile.com/b/5l1Ezu/Lr7C)

[Dabney, Jesse, Michael Knapp, Isabelle Glocke, Marie-Theres Gansauge, Antje Weihmann, Birgit Nickel, Cristina Valdiosera, et al. 2013. “Complete Mitochondrial Genome Sequence of a Middle Pleistocene Cave Bear Reconstructed from Ultrashort DNA Fragments.” *Proceedings of the National Academy of Sciences of the United States of America* 110 (39): 15758–63.](http://paperpile.com/b/5l1Ezu/fwyv)

[Damgaard, Peter B., Ashot Margaryan, Hannes Schroeder, Ludovic Orlando, Eske Willerslev, and Morten E. Allentoft. 2015. “Improving Access to Endogenous DNA in Ancient Bones and Teeth.” *Scientific Reports* 5 (June): 11184.](http://paperpile.com/b/5l1Ezu/tQeg)

[Jørsboe, Emil, Kristian Hanghøj, and Anders Albrechtsen. 2017. “fastNGSadmix: Admixture Proportions and Principal Component Analysis of a Single NGS Sample.” *Bioinformatics*  33 (19): 3148–50.](http://paperpile.com/b/5l1Ezu/fSBO)

[Korneliussen, Thorfinn Sand, Anders Albrechtsen, and Rasmus Nielsen. 2014. “ANGSD: Analysis of Next Generation Sequencing Data.” *BMC Bioinformatics* 15 (November): 356.](http://paperpile.com/b/5l1Ezu/MNSi)

[Li, H., and R. Durbin. 2009. “Fast and Accurate Short Read Alignment with Burrows–Wheeler Transform.” *Bioinformatics* .](http://paperpile.com/b/5l1Ezu/s83N) <https://academic.oup.com/bioinformatics/article-abstract/25/14/1754/225615>[.](http://paperpile.com/b/5l1Ezu/s83N)

[Li, Heng, Bob Handsaker, Alec Wysoker, Tim Fennell, Jue Ruan, Nils Homer, Gabor Marth, Goncalo Abecasis, Richard Durbin, and 1000 Genome Project Data Processing Subgroup. 2009. “The Sequence Alignment/Map Format and SAMtools.” *Bioinformatics*  25 (16): 2078–79.](http://paperpile.com/b/5l1Ezu/mlE3)

[Lindgreen, Stinus. 2012. “AdapterRemoval: Easy Cleaning of next-Generation Sequencing Reads.” *BMC Research Notes* 5 (July): 337.](http://paperpile.com/b/5l1Ezu/kZDN)

[McKenna, Aaron, Matthew Hanna, Eric Banks, Andrey Sivachenko, Kristian Cibulskis, Andrew Kernytsky, Kiran Garimella, et al. 2010. “The Genome Analysis Toolkit: A MapReduce Framework for Analyzing next-Generation DNA Sequencing Data.” *Genome Research* 20 (9): 1297–1303.](http://paperpile.com/b/5l1Ezu/2nmN)

[Meisner, Jonas, and Anders Albrechtsen. 2018. “Inferring Population Structure and Admixture Proportions in Low-Depth NGS Data.” *Genetics* 210 (2): 719–31.](http://paperpile.com/b/5l1Ezu/RWIH)

[Meyer, Matthias, and Martin Kircher. 2010. “Illumina Sequencing Library Preparation for Highly Multiplexed Target Capture and Sequencing.” *Cold Spring Harbor Protocols* 2010 (6): db.prot5448.](http://paperpile.com/b/5l1Ezu/OXXO)

[Schubert, Mikkel, Luca Ermini, Clio Der Sarkissian, Hákon Jónsson, Aurélien Ginolhac, Robert Schaefer, Michael D. Martin, et al. 2014. “Characterization of Ancient and Modern Genomes by SNP Detection and Phylogenomic and Metagenomic Analysis Using PALEOMIX.” *Nature Protocols* 9 (5): 1056–82.](http://paperpile.com/b/5l1Ezu/6Shs)

[Skotte, Line, Thorfinn Sand Korneliussen, and Anders Albrechtsen. 2013. “Estimating Individual Admixture Proportions from next Generation Sequencing Data.” *Genetics* 195 (3): 693–702.](http://paperpile.com/b/5l1Ezu/9HMx)

[Skovrind, Mikkel, Jose Alfredo Samaniego Castruita, James Haile, Eve C. Treadaway, Shyam Gopalakrishnan, Michael V. Westbury, Mads Peter Heide-Jørgensen, Paul Szpak, and Eline D. Lorenzen. 2019. “Hybridization between Two High Arctic Cetaceans Confirmed by Genomic Analysis.” *Scientific Reports* 9 (1): 7729.](http://paperpile.com/b/5l1Ezu/PJ1W)
