# Supplementary material for: Skull ecomorphological variation of narwhals (Monodon monoceros, Linnaeus 1758) and belugas (Delphinapterus leucas, Pallas 1776) reveals phenotype of their hybrids
Source: PLoS One. 2022 Aug 12;17(8):e0273122. doi: 10.1371/journal.pone.0273122 (PMC9374245; doi:10.1371/journal.pone.0273122)
Supplement: S4 File — Procrustes ANOVA to test for allometry in (A) 49 sexed narwhals and (B) 24 sexed belugas. Significance is highlighted in bold. (DOCX) [file pone.0273122.s009.docx]

|  |  | **Df** | **SS** | **MS** | **Rsq** | **F** | **Z** | **P** |
| --- | --- | --- | --- | --- | --- | --- | --- | --- |
| **A)**  **Narwhal**  **Allometry** | **CS** | 1 | 0.0598 | 0.0598 | 0.2713 | 17.83 | 4.0922 | **0.001** |
|  | **Sex** | 1 | 0.0039 | 0.0039 | 0.0177 | 1.1669 | 0.6867 | 0.251 |
|  | **CS:Sex** | 1 | 0.0057 | 0.0057 | 0.0262 | 1.7226 | 2.2404 | **0.018** |
|  | **Residuals** | 45 | 0.1511 | 0.0033 | 0.6847 |  |  |  |
|  | **Total** | 48 | 0.2207 |  |  |  |  |  |
| **Narwhal**  **Size** | **Sex** | 1 | 0.1148 | 0.1148 | 0.1964 | 11.492 | 2.5626 | **0.004** |
|  | **Residuals** | 47 | 0.4698 | 0.0099 | 0.8035 |  |  |  |
|  | **Total** | 48 |  |  |  |  |  |  |
| **B)**  **Beluga**  **Allometry** | **CS** | 1 | 0.0318 | 0.0318 | 0.2800 | 8.7538 | 3.9438 | **0.001** |
|  | **Sex** | 1 | 0.0042 | 0.0042 | 0.0373 | 1.1688 | 0.6586 | 0.260 |
|  | **CS:Sex** | 1 | 0.0048 | 0.0048 | 0.0427 | 1.3373 | 1.0755 | 0.137 |
|  | **Residuals** | 20 | 0.0727 | 0.0036 | 0.6398 |  |  |  |
|  | **Total** | 23 |  |  |  |  |  |  |
| **Beluga**  **Size** | **Sex** | 1 | 28750 | 28750 | 0.0973 | 2.3736 | 1.059 | 0.153 |
|  | **Residuals** | 22 | 66478 | 12113 | 0.9026 |  |  |  |
|  | **Total** | 23 |  |  |  |  |  |  |

**S4 File. Procrustes ANOVA to test for A) allometry of 49 sexed narwhals and B) 24 sexed belugas. Significance is highlighted in bold.**
